# Dopant-additive synergism enhances perovskite solar modules

**DOI:** 10.1038/s41586-024-07228-z

**Published:** 2024-03-04

**Authors:** Bin Ding, Yong Ding, Jun Peng, Jan Romano-deGea, Lindsey E. K. Frederiksen, Hiroyuki Kanda, Olga A. Syzgantseva, Maria A. Syzgantseva, Jean-Nicolas Audinot, Jerome Bour, Song Zhang, Tom Wirtz, Zhaofu Fei, Patrick Dörflinger, Naoyuki Shibayama, Yunjuan Niu, Sixia Hu, Shunlin Zhang, Farzaneh Fadaei Tirani, Yan Liu, Guan-Jun Yang, Keith Brooks, Linhua Hu, Sachin Kinge, Vladimir Dyakonov, Xiaohong Zhang, Songyuan Dai, Paul J. Dyson, Mohammad Khaja Nazeeruddin

**Affiliations:** 1 Institute of Chemical Sciences and Engineering, École Polytechnique Fédérale de Lausanne (EPFL), Lausanne, Switzerland; 2 State Key Laboratory of Alternate Electrical Power System with Renewable Energy Sources, North China Electric Power University, Beijing, P. R. China; 3 Institute of Functional Nano & Soft Materials (FUNSOM), Jiangsu Key Laboratory of Advanced Negative Carbon Technologies, Joint International Research Laboratory of Carbon-Based Functional Materials and Devices, Soochow University, Suzhou, P. R. China; 4 Department of Chemistry, Lomonosov Moscow State University, Moscow, Russia; 5 Advanced Instrumentation for Nano-Analytics (AINA), Materials Research and Technology (MRT) Department, Luxembourg Institute of Science and Technology (LIST), Belvaux, Luxembourg; 6 State Key Laboratory of Magnetic Resonance and Atomic and Molecular Physics, Innovation Academy for Precision Measurement Science and Technology, Chinese Academy of Sciences, Wuhan, P. R. China; 7 Institute of Physics, Julius Maximilian University of Würzburg, Würzburg, Germany; 8 Faculty of Biomedical Engineering, Graduate School of Engineering, Toin University of Yokohama, Yokohama, Japan; 9 Key Laboratory of Photovoltaic and Energy Conservation Materials, CAS, Institute of Solid-State Physics, Hefei Institutes of Physical Science, Chinese Academy of Sciences, Hefei, P. R. China; 10 Materials Characterization and Preparation Center, Southern University of Science and Technology, Shenzhen, P. R. China; 11 State Key Laboratory for Mechanical Behavior of Materials, School of Materials Science and Engineering, Xi’an Jiaotong University, Xi’an, P. R. China; 12 Materials Engineering Division, Toyota Technical Centre, Toyota Motor Europe, Zaventem, Belgium

**Keywords:** Solar cells, Solar cells

## Abstract

Perovskite solar cells (PSCs) are among the most promising photovoltaic technologies owing to their exceptional optoelectronic properties^1,2^. However, the lower efficiency, poor stability and reproducibility issues of large-area PSCs compared with laboratory-scale PSCs are notable drawbacks that hinder their commercialization^3^. Here we report a synergistic dopant-additive combination strategy using methylammonium chloride (MACl) as the dopant and a Lewis-basic ionic-liquid additive, 1,3-bis(cyanomethyl)imidazolium chloride ([Bcmim]Cl). This strategy effectively inhibits the degradation of the perovskite precursor solution (PPS), suppresses the aggregation of MACl and results in phase-homogeneous and stable perovskite films with high crystallinity and fewer defects. This approach enabled the fabrication of perovskite solar modules (PSMs) that achieved a certified efficiency of 23.30% and ultimately stabilized at 22.97% over a 27.22-cm^2^ aperture area, marking the highest certified PSM performance. Furthermore, the PSMs showed long-term operational stability, maintaining 94.66% of the initial efficiency after 1,000 h under continuous one-sun illumination at room temperature. The interaction between [Bcmim]Cl and MACl was extensively studied to unravel the mechanism leading to an enhancement of device properties. Our approach holds substantial promise for bridging the benchtop-to-rooftop gap and advancing the production and commercialization of large-area perovskite photovoltaics.

## Abstract (web summary)

A synergistic dopant-additive combination strategy using methylammonium chloride as the dopant and a Lewis-basic ionic-liquid additive is shown to enable the fabrication of perovskite solar modules achieving record certified performance and long-term operational stability.

## Main

Formamidinium-based lead perovskites (FAPbI_3_) show optimal bandgap absorption, but the formation of their photoactive ‘black phase’ (or α phase) is energetically unfavourable and the resulting perovskite thin films (PTFs) suffer from low stability owing to low crystallinity^4^. Dopants that are incorporated into the perovskite structure^5–8^ modulate the optical and thermodynamic properties of FAPbI_3_. Among these dopants, MACl reduces perovskite formation energy, stabilizes the black-phase perovskite and favours crystal growth in preferential orientations^9^. However, unfavourable intermediate phases that negatively affect crystallinity, phase purity and performance are not suppressed simply by doping with MACl. Further tuning of perovskite fabrication by incorporating additives such as ionic liquids results in perovskites with improved device efficiency and stability through passivation of defects, enhancement of crystallinity and phase purity or suppression of film decomposition^10–14^. Our previous work on ionic liquids has demonstrated that imidazolium-based cations with Lewis-basic nitrile functional groups with highly electronegative Cl^−^ anions can greatly enhance PTF stability and efficiency^14^. In this work, the structurally optimized ionic liquid [Bcmim]Cl was combined with MACl for the formation of high-quality Cs_0.05_MA_0.05_FA_0.90_Pb(I_1−*x*_Cl_*x*_)_3_ perovskite films with uniform, well-oriented grains and improved phase purity, resulting in the fabrication of extremely efficient and stable PSCs and PSMs. The interaction between [Bcmim]Cl and MACl and their respective roles was systematically investigated to explain the underlying mechanisms inducing their positive effects.

## [Bcmim]Cl/MACl-containing PSC performance

**Fig. 1:**
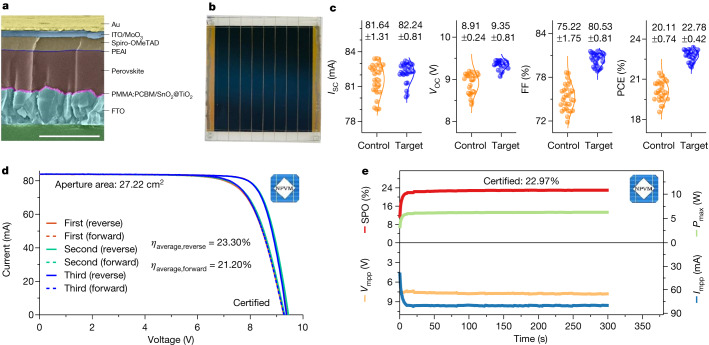
PSM structure and photovoltaic performance of the control and target PSMs. **a**, Cross-sectional SEM image of the target device. Scale bar, 1 μm. **b**, Picture of one PSM. **c**, Distribution of short-circuit current (*I*_SC_), *V*_OC_, FF and PCE. **d**, Current–voltage (*I–V*) curves of three consecutive tests from forward and reverse scans for the certified PSM. **e**, Stabilized power output (SPO) performance of the certified PSM.

N-i-p-structured PSCs were fabricated using Cs_0.05_MA_0.05_FA_0.90_Pb(I_1−*x*_Cl_*x*_)_3_ PTFs (Fig. 1a). We evaluated the effects on PSC efficiency of an imidazolium-based ionic liquid series with Cl^−^ anions as perovskite additives in combination with MACl. Among the investigated ionic liquids, only [Bcmim]Cl markedly enhanced the efficiency of PSCs (Supplementary Fig. 1). Subsequently, we explored a series of ionic liquids pairing [Bcmim]^+^ with different anions, including ${{\rm{BF}}}_{4}^{-}$, ${{\rm{PF}}}_{6}^{-}$, I^−^ and SCN^−^. Our findings revealed that all of the [Bcmim]^+^-based ionic liquids substantially increased the efficiency of PSCs (Supplementary Fig. 2), but [Bcmim]Cl still yielded the highest efficiency. Consequently, we focused our research on the [Bcmim]Cl/MACl system. The concentrations of MACl and [Bcmim]Cl were optimized (0–60 mol% and 0–1.4 mol%, respectively (Supplementary Figs. 3 and 4)). The PSCs containing 20 mol% MACl and 0.6 mol% [Bcmim]Cl (‘target’) achieved an average power conversion efficiency (PCE) of 25.21 ± 0.16% with a short-circuit current density (*J*_SC_) of 25.71 ± 0.06 mA cm^−2^, an open-circuit voltage (*V*_OC_) of 1.17 ± 0.01 V and a fill factor (FF) of 83.97 ± 0.37%. These values are much higher than those of the devices with 20 mol% MACl but without [Bcmim]Cl (‘control’). The improvement in PCE was primarily attributed to enhanced FF and *V*_OC_, whereas the *J*_SC_ exhibited little difference across the groups (Supplementary Fig. 4).

PSMs consisting of eight subcells connected in series were prepared (Fig. 1b). The target PSMs exhibited greatly improved performance, achieving an average PCE of 22.78 ± 0.42% over the control PSMs (average PCE of 20.11 ± 0.74%; Fig. 1c) on a 27.22-cm^2^ aperture area. Independent testing conducted by the Chinese National PV Industry Measurement and Testing Center (NPVM), a reputable certification body^15^, showed the highest certified PSM performance (Supplementary Table 1). The certified efficiencies of the target PSM were 23.30% ± 0.01% (reverse) and 21.20% ± 0.14% (forward; Fig. 1d and Supplementary Fig. 5). After about 300 s of maximum power point (MPP) tracking, the target PSM demonstrated an average stabilized power output efficiency of 22.50%, eventually stabilizing at 22.97% (Fig. 1e and Supplementary Fig. 6). To address issues associated with scalability and transferability inherent to the spin-coating fabrication method, a blade-coating approach was used, showing that the [Bcmim]Cl/MACl-based PSM still achieves high efficiencies (21.56 ± 0.442%) (Supplementary Fig. 7) and demonstrating its viability for large-scale manufacturing.

## [Bcmim]Cl-mediated PPS stabilization

**Fig. 2:**
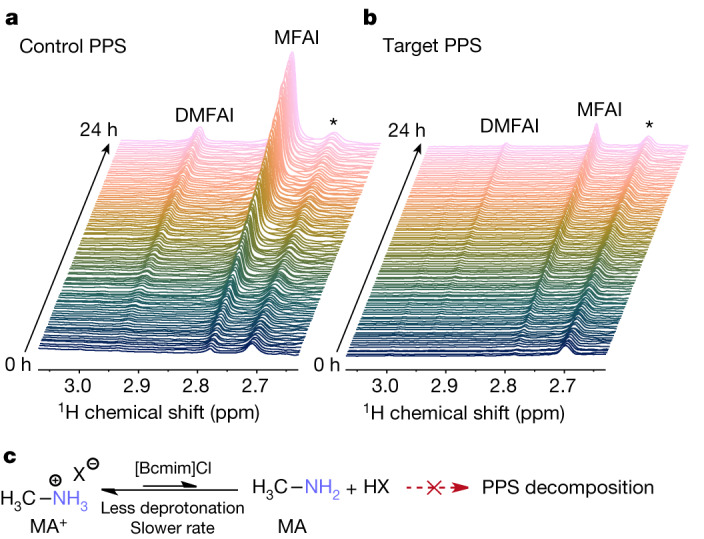
^1^H NMR spectra of the PPS degradation at 60 °C for 24 h. **a**, Control. **b**, Target. Key peaks from MFAI (2.78 ppm) and DMFAI (2.94 ppm) are highlighted. Spectra were recorded every 15 min in a mixture of d_6_-DMSO:d_7_-DMF with the ratio used for PSC fabrication and the intensity is normalized to the residual solvent peak (*). **c**, Mechanism of the [Bcmim]Cl-mediated stabilization of the PPS.

The PPSs used for PSM fabrication suffer from short shelf lives^16,17^ and their decomposition detrimentally affects both PTF crystallinity and device repeatability^18^. The decomposition of both mixed MA^+^/FA^+^ perovskites and their constituent PPSs has been previously studied^19–21^ (Supplementary Fig. 8). The interaction between [Bcmim]Cl and MACl, as well as its impact on PPS stability, were investigated by examining species present in the PPS as a function of temperature and time using in operando nuclear magnetic resonance (NMR) spectroscopy. Mixed FA^+^/MA^+^ PPSs decompose by means of a previously reported condensation reaction, with singly methylated FAI (*N*-methylformamidinium iodide, MFAI) and doubly methylated FAI (*N,N*-dimethylformamidinium iodide, DMFAI) as the primary products, giving rise to peaks at 2.78 ppm and 2.94 ppm, respectively, in the ^1^H NMR spectra of the studied system^22,23^ (Supplementary Fig. 9). PPS stability was determined by monitoring the appearance of these species in ^1^H NMR spectra of the PPSs at varying temperatures. The ^1^H NMR peaks corresponding to MFAI and DMFAI first appear at 30 °C and 60 °C, respectively, for the control PPS. However, in the presence of 0.6 mol% [Bcmim]Cl (target PPS), condensation reaction products are only observed at higher temperatures, that is, 55 °C for MFAI and 75 °C for DMFAI (Supplementary Fig. 10). Further kinetic studies of PPS stability were conducted at 25 °C and 60 °C over a period of 24 h, showing the increased stability of MA^+^ in the target PPS (Fig. 2 and Supplementary Fig. 11). Slower decomposition of MA^+^ in the presence of [Bcmim]Cl is also supported by a smaller downfield (to higher δ values) peak shift in the ^207^Pb NMR spectra of the target PPS both before and after 24 h (Supplementary Fig. 12a,b) compared with the control PPS (Supplementary Fig. 12c,d). In this system, changes in ^207^Pb peak chemical shift indicate an increase in free halide content, linked to extent of decomposition. Notably, PTFs fabricated from target PPSs have the same morphology regardless of whether the PPS was freshly prepared or aged for 10 days, whereas the control PTFs show notable symptoms of degradation when not made from a fresh PPS (Supplementary Fig. 13). By considerably slowing PPS decomposition, [Bcmim]Cl leads to high-quality PTFs and, as a result, highly efficient and stable PSMs.

**Fig. 3:**
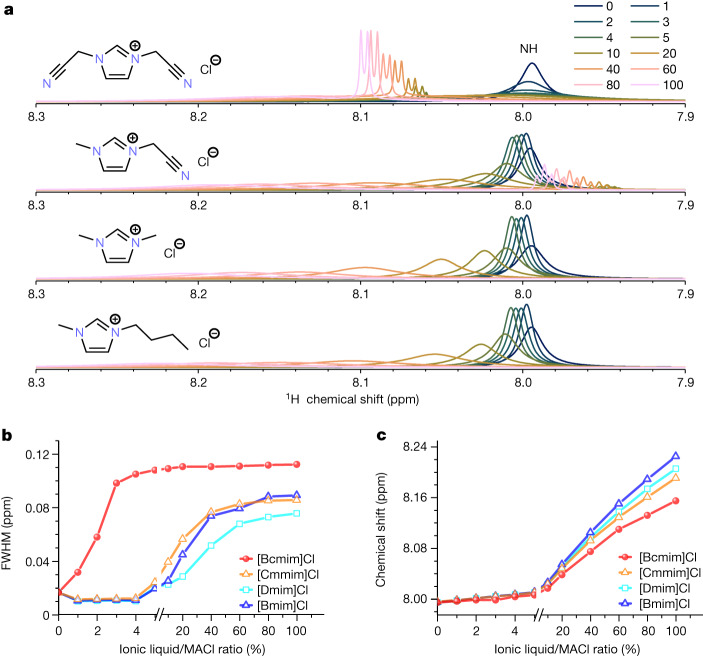
NMR spectra of the interaction between [Bcmim]Cl and MACl. **a**, ^1^H NMR spectra of MACl solution as a function of concentration for four ionic liquids: [Bcmim]Cl, 1-cyanomethyl-3-methylimidazolium chloride ([Cmmim]Cl), 1,3-dimethylimidazolium chloride ([Dmim]Cl) and 1-butyl-3-methylimidazolium chloride ([Bmim]Cl). Full width at half maximum (FWHM) shift (**b**) and chemical shift of the MA^+^ NH peak as a function of ionic liquid/MACl concentration ratio (**c**).

The improved stability and efficiency of the target PSCs highlight the synergistic effect from combining [Bcmim]Cl and MACl in the PTF. To clarify potential stabilization mechanisms and/or interactions between MACl and [Bcmim]Cl that might contribute to the observed synergism, several model NMR spectroscopic experiments were carried out on the PPS (Fig. 3a). Adding [Bcmim]Cl (0.01–1 eq., 0.2–20 mol% relative to the operational concentration of PbI_2_) to a MACl solution results in substantial broadening (from 0.015 ppm to 0.1 ppm; Fig. 3b) of the MA^+^ cation NH peak (7.99 ppm) in the ^1^H NMR spectra. Furthermore, as the concentration of [Bcmim]Cl increases, this signal shifts from 7.99 ppm to 8.23 ppm (Fig. 3c). Replacing MACl by MAI results in identical changes to the ^1^H NMR spectra (Supplementary Fig. 14a), indicating that these effects are intrinsic to the MA^+^ cation. The addition of other ionic liquids previously used as additives^10,14^ (Fig. 3a) or LiCl (Supplementary Fig. 14b) with MACl also results in a broadening of the peak, but only the combinations with [Bcmim]Cl show a notable effect at relevant fabrication concentrations (0.6 mol% relative to operational concentration of PbI_2_) for the real PPS and PTF systems (Fig. 3b).

The observed downfield shift of the NH peak in the ^1^H NMR spectrum with the addition of all ionic liquids tested (Fig. 3c) is indicative of hydrogen bonding, which has been shown to improve PTF stability and efficiency^24^. Efficient passivation of PSCs containing bis(cyanomethyl)imidazolium bis(trifluoromethylsulfonyl)imide ([Bcmim]TFSI), which establishes an extended hydrogen bond network to improve the stability of the prepared PTFs, has been previously reported^13^. [Bcmim]Cl was found to be a stronger hydrogen bond acceptor than [Bcmim]TFSI (Supplementary Fig. 15) using an established method^25^ and may induce further stabilizing effects.

Peak broadening demonstrates that the NH protons are engaging in chemical exchange (see Supplementary Note 1 and Supplementary Table 2). The interaction between these protons and other exchangeable protons in the PPS, such as the one in [Bcmim]Cl, accelerates nuclear relaxation of the NH proton, inducing peak broadening in the ^1^H NMR spectra. Imidazolium-based ionic liquids can act as weak Brønsted acids through the proton in the 2-position. As confirmed by pH measurements (Supplementary Table 3), the electron-withdrawing nitrile-functionalized side chains in [Bcmim]Cl increase the acidity of this ionic liquid relative to the others investigated in this work. NMR exchange spectroscopy (EXSY), used to detect proton exchange, indicates that the NH protons of MACl and the acidic 2-proton of the [Bcmim]^+^ cation exchange in solution (Supplementary Fig. 16a). Notably, this exchange process is also visible in the EXSY NMR spectra of the target PPS (Supplementary Fig. 16b) but not observed for [Bcmim]^+^ when paired with other anions such as ${{\rm{BF}}}_{4}^{-}$, ${{\rm{PF}}}_{6}^{-}$, I^−^ and SCN^−^ (Supplementary Fig. 17), emphasizing the uniqueness of the Cl^−^ salt. The observed proton exchange and reduced relaxation indicate that the deprotonation of MA^+^ to methylamine is inhibited (Fig. 2c), deterring the condensation reaction between methylamine and FA^+^ (Supplementary Fig. 9) and stabilizing the PPS.

## [Bcmim]Cl/MACl-based PTF characteristics

The impact of [Bcmim]Cl on PTFs was investigated by preparing films from PPSs containing 20 mol% MACl and 0–1.4 mol% [Bcmim]Cl. X-ray diffraction (XRD) patterns of the PTFs are all comparable, with the (110) peak intensity increasing with increasing [Bcmim]Cl content (Supplementary Fig. 18a). As evidenced by rocking curves and grazing-incidence wide-angle X-ray scattering (GIWAXS) (Supplementary Fig. 18b–d), the addition of [Bcmim]Cl promotes crystal growth preferentially in the (110) and (220) orientations. Scanning electron microscopy (SEM) images reveal increased average grain size in perovskite crystals with increasing [Bcmim]Cl content (Supplementary Fig. 18e). Combining MACl and [Bcmim]Cl not only improves PTF crystallinity and promotes crystal growth along preferred orientations but is also applicable to [Bcmim]-based ionic liquids with other anions (Supplementary Fig. 19). Furthermore, the target has a much higher steady-state photoluminescence (PL) mapping intensity than the control PTF, consistent with the PL quantum yield (PLQY) and time-resolved PL (TRPL) spectra (Supplementary Fig. 20). Increased PLQY, TRPL lifetimes and charge-carrier diffusion in target PTFs indicate suppressed trap-assisted nonradiative recombination (Supplementary Table 4), confirming the improved *V*_OC_ (refs. ^26,27^). Therefore, combining [Bcmim]Cl and MACl reduces PTF defects and increases charge-carrier lifetime, which both improve device performance.

**Fig. 4:**
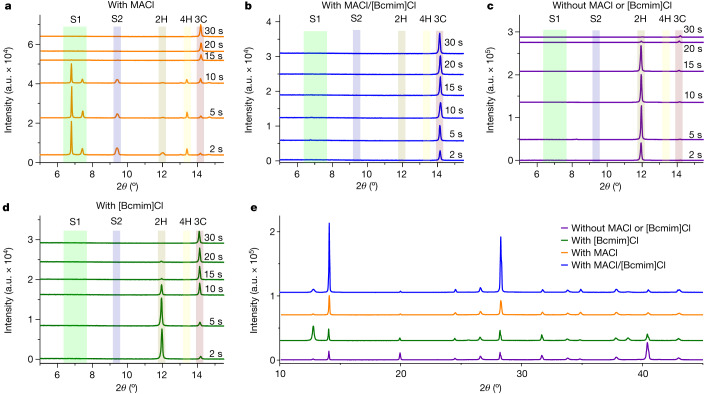
XRD patterns of the PTFs as a function of annealing time. **a**, XRD patterns of the control PTF with only MACl as a function of annealing (100 °C) time. The peaks at 6.79° and 7.41° correspond to solvate phase S1 and the peak at 9.41° corresponds to solvate phase S2 (ref. ^31^). The peaks located at 11.99°, 13.39° and 14.15° correspond to the 2H phase (δ phase), hexagonal phase (4H) and 3C phase (α phase), respectively^32^. **b**, XRD patterns of the target PTF with both [Bcmim]Cl and MACl as a function of annealing (100 °C) time. **c**, XRD patterns of the PTF without MACl or [Bcmim]Cl as a function of annealing (100 °C) time. **d**, XRD patterns of the PTFs with only [Bcmim]Cl as a function of annealing (100 °C) time. **e**, XRD patterns of the PTFs after annealing at 100 °C for 60 min and at 150 °C for 10 min. The peak located at 12.70° corresponds to the (001) peak of PbI_2_. a.u., arbitrary units.

To determine how MACl and [Bcmim]Cl improve PTF quality, the intermediate phase transition during the annealing process from precursor PTF to crystalline PTF was monitored by XRD. Unfavourable intermediate phases (see Fig. 4 legend for peak assignments) are present in the control PTFs during annealing (Fig. 4a). The unfavourable intermediate phase peaks decrease in intensity with prolonged annealing time and disappear after 30 s. Meanwhile, the intensity of the black-phase perovskite peak (3C or α phase, 14.15°) increases with extended annealing time, becoming the dominant phase after 30 s. By contrast, the unfavourable intermediate phases are initially absent in the target PTFs. The XRD pattern is instead dominated by the black-phase perovskite peak (Fig. 4b), which increases in intensity with prolonged annealing time. PPSs that contain neither MACl nor [Bcmim]Cl crystallize by means of a 2H → 3C sequence (Fig. 4c), whereas adding [Bcmim]Cl alone considerably suppresses the 4H, S1 and S2 phases, inducing the 2H → 3C transition after less annealing time (Fig. 4d). The black-phase peak for the target PTF is more intense than for the control PTF and the PTF with MACl alone (8.27 and 3.66 times higher, respectively), indicating that only the synergism between MACl and [Bcmim]Cl substantially boosts PTF crystallinity (Fig. 4e). SEM images show that increasing MACl concentration enhances PTF crystallinity and grain size (Supplementary Figs. 21 and 22), but ≥30 mol% MACl induces excess pinhole formation (Supplementary Fig. 22). However, PTFs containing [Bcmim]Cl and high MACl concentrations demonstrate suppressed pinhole formation, maintaining large grains with greater uniformity (Supplementary Fig. 23).

**Fig. 5:**
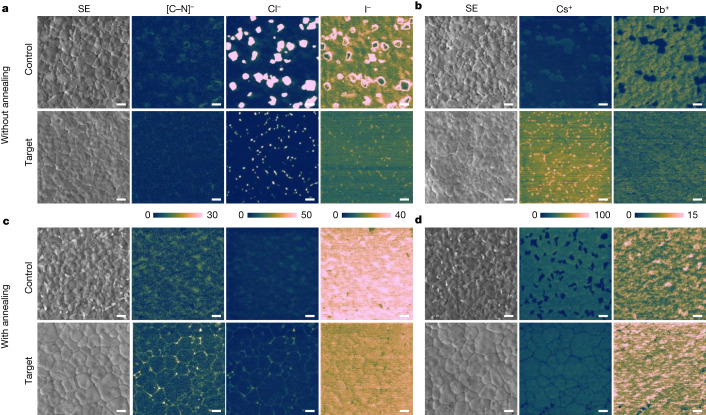
Secondary electron images with corresponding compositional mapping of PTFs by HIM-SIMS imaging. Negative-mode (**a**) and positive-mode (**b**) SIMS polarity imaging of PTFs without annealing. Negative-mode (**c**) and positive-mode (**d**) SIMS polarity imaging of PTFs annealed at 100 °C for 60 min then at 150 °C for 10 min. SE, secondary electron. Scale bars, 1 µm.

Phase distribution in the target and control PTFs, before and after annealing, was investigated using helium ion microscopy coupled with secondary ion mass spectrometry (HIM-SIMS). Unannealed control PTFs exhibit considerable MACl clustering, as evidenced by the co-localized concentrations of [C–N]^−^ and Cl^−^ ions (Fig. 5a). Similarly, Cs^+^ aggregation is observed, as evidenced by areas enriched with Cs^+^ and depleted of Pb^+^ (Fig. 5b), which might be attributed to the low solubility of caesium salts in the PPS^28^. By contrast, unannealed target PTFs have a more homogeneous phase distribution (Fig. 5c), attributable to the presence of [Bcmim]Cl. After annealing, both PTFs exhibit improved phase uniformity. The Cs-based aggregates melt and disappear (Fig. 5d), in accordance with previous reports that annealing at 150 °C converts Cs-based aggregates into perovskite phases and eliminates local inhomogeneities^28^. However, the annealed target PTFs exhibit less Cs^+^ depletion. Depth profiling of the target PTF using time-of-flight SIMS (ToF-SIMS) reveals that [Bcmim]^+^ is uniformly distributed across the grain boundaries (Supplementary Fig. 24). Adding [Bcmim]Cl to the PPS maintains the large MACl-induced PTF grain sizes while improving crystal uniformity and quality by encouraging direct transformation to black-phase perovskite and even distribution of MACl and other components in the PTF.

High-resolution X-ray photoelectron spectroscopy spectra of PTFs incorporating increasing [Bcmim]Cl concentrations show marked Pb 4f peak shifts to higher energy, indicating that [Bcmim]Cl interacts with Pb^2+^ in the PTF (Supplementary Fig. 25). The [Bcmim]Cl adsorption energies on various perovskite surfaces were calculated using density functional theory (DFT) and show that both the nitrile groups in [Bcmim]^+^ and Cl^−^ are able to interact with Pb^2+^ and occupy halide vacancies (Supplementary Fig. 26). The passivation of undercoordinated Pb^2+^ sites has also been proposed to stabilize PTFs^10^. Solution ^207^Pb NMR spectra of the target and control PPSs further support the presence of interactions between [Bcmim]^+^ and Pb^2+^ ions (Supplementary Fig. 27). Because [Bcmim]Cl is distributed throughout the PTF (Supplementary Fig. 24b), it is plausible that it passivates undercoordinated sites at the grain boundaries within the PTF. However, the nature of this interaction is unlikely to be the formation of new Pb-N dative bonds as coordination of [Bcmim]^+^ nitrile groups to Pb^2+^ is not evident in single-crystal structures derived from model reactions (Supplementary Fig. 28) or DFT calculation results (Supplementary Fig. 26).

Dynamic light scattering (DLS) measurements of the control PPS reveal two peaks with average diameters of 1.65 and 4,380 nm, respectively. A further peak at 600 nm is present for PPS containing [Bcmim]Cl (Supplementary Fig. 29), suggesting that [Bcmim]Cl may induce the formation of a new aggregate form. In fact, a model reaction between [Bcmim]Cl and PbI_2_ in DMF, intended to mimic reactivity and interactions in the grain boundaries, yields a 1D intermediate with the empirical formula [Bcmim]_4_Pb_3_Cl_2_I_6_·2DMF (Supplementary Fig. 28). It is plausible that, once formed, these 1D salts act as nucleation sites, promoting perovskite crystallization. Compared with [Bcmim]Cl, the non-nucleophilic TFSI^−^ anion in [Bcmim]TFSI (ref. ^13^) is less likely to participate in counterion exchange processes that lead to the formation of these 1D salts (Supplementary Fig. 28e).

Furthermore, as solvents evaporate during annealing, MACl and [Bcmim]Cl may react with FAI and PbI_2_ (as ${{\rm{PbI}}}_{3}^{-}$) to form a eutectic ionic liquid^29^. As demonstrated by polarized light optical microscopy (Supplementary Fig. 30a–d), crystal growth from eutectic ionic liquid mixtures is substantially decelerated, allowing the formation of large, homogeneous, well-ordered crystals. Incorporating [Bcmim]Cl into the PPS causes more small monodispersed aggregates to form. However, at high concentrations, [Bcmim]Cl still promotes nucleation, as evidenced by the huge number of crystal seeds (Supplementary Fig. 30e), but also inhibits crystal growth, limiting the use of higher [Bcmim]Cl concentrations. [Bcmim]Cl promotes the homogeneous distribution of MACl, increasing crystal nucleation while reducing the growth rate of individual crystals. After thermal treatment, the aggregation of these particles results in the crystallization of a more homogeneous film with larger grain sizes and fewer defects. Adding both MACl and [Bcmim]Cl to the PPS has a synergistic effect on the PTF, enhancing film crystallinity, suppressing unfavourable intermediate phases, reducing defects and improving quantum yield and PL lifetime.

## Long-term operational stability

Perovskite instability, caused by heat, oxygen, moisture, potential bias and ultraviolet (UV) light, is a substantial challenge as decomposition decreases photovoltaic activity^30^. Thermal ageing of the control PTF at 60 ± 5 °C for 600 h resulted in a gradual redshift of the UV–Vis absorption edge (Supplementary Fig. 31). XRD patterns of the control PTF show a gradual increase in PbI_2_ content throughout the ageing process (Supplementary Fig. 32). By contrast, the target PTF exhibits a smaller redshift than the control PTF (Δ*λ* = 2.41 versus 9.76 nm; Supplementary Fig. 31), indicating improved thermal stability. After 600 h of ageing, XRD patterns of the target PTF show retention of the desirable black phase and suppression of PbI_2_ formation (Supplementary Fig. 32). This suggests that the combination not only stabilizes the PPS but mitigates PTF degradation and improves intrinsic stability by suppressing thermal decomposition.

The stability of PSMs containing [Bcmim]Cl and MACl was evaluated using two International Summit on Organic Photovoltaic Stability (ISOS) protocols^30^. Under continuous one-sun illumination at room temperature (ISOS-L-1), the target PSM retained 94.66% of its initial efficiency after 1,000 h and the control PSM maintained only 84.53% (Supplementary Fig. 33a). Furthermore, when heated (65 °C) under one-sun illumination (ISOS-L-2), the target PSM maintained 87.19% of its original efficiency compared with only 75.80% for the control PSM (Supplementary Fig. 33b). The retention of efficiency highlights that the combination of [Bcmim]Cl and MACl effectively improves PSM long-term operational stability.

## Conclusions

Highly efficient and stable perovskite-based photovoltaics were obtained with a synergistic dopant-additive combination strategy. [Bcmim]Cl and MACl interact to stabilize the PPS by inhibiting the condensation reaction between MACl and FAI, resulting in solutions with longer lifetimes, as well as high-quality films with large and oriented grain sizes and better phase purity. The resulting PSCs and PSMs show that combining MACl and [Bcmim]Cl improves efficiency and stability while allowing scaled-up production. The underlying molecular basis for the improvement caused by combining MACl with [Bcmim]Cl was established along with its impact on PPS stability and the resulting positive effects. We expect these findings to contribute to the large-scale production and commercialization of perovskite-based photovoltaic technologies.

## Methods

### Materials

Titanium(IV) chloride (TiCl_4_, for synthesis), tin(II) chloride (SnCl_2_, reagent grade, 98%), lithium bis(trifluoromethylsulfonyl)imide (99.95% trace metals basis), tris(2-(*1H*-pyrazol-1-yl)-4-tertbutylpyridine)cobalt(III) (FK209), polymethyl methacrylate (PMMA), [6,6]-phenyl-*C*61-butyric acid methyl ester (PCBM), caesium chloride (99.9%), imidazolium chloride ([Im]Cl), 1,3-dimethylimidazolium chloride ([Dmim]Cl), 1-butyl-3-methylimidazolium chloride ([Bmim]Cl), 1-cyanomethyl-3-methylimidazolium chloride ([Cmmim]Cl), trifluoroacetic acid (99%), molybdenum(VI) oxide (MoO_3_, 99.97% trace metals basis) and HCl (4 M in 1,4-dioxane) were purchased from Sigma-Aldrich. Water-free PEDOT:complex HTL Solar 3 in toluene was obtained from Ossila (product no. M125). Tris(pentafluorophenyl)borane (97%) was obtained from Shanghai Aladdin Biochemical Technology Co., Ltd. Methylammonium iodide (MAI), MACl, formamidinium iodide (FAI) and phenethylammonium iodide (PEAI) were purchased from GreatCell Solar Ltd. Lead(II) iodide (PbI_2_, 99.99%, trace metals basis) was purchased from Tokyo Chemical Industry Co., Ltd. (TCI). [Bcmim]Cl was prepared according to a literature method^33^. 1,3-bis(cyanomethyl)imidazolium bis(trifluoromethylsulfonyl)imide ([Bcmim]TFSI), 1,3-bis(cyanomethyl)imidazolium tetrafluoroborate ([Bcmim]BF_4_), bis(cyanomethyl)imidazolium iodide ([Bcmim]I), bis(cyanomethyl)imidazolium hexafluorophosphate ([Bcmim]PF_6_) and bis(cyanomethyl)imidazolium thiocyanate ([Bcmim]SCN) were prepared from [Bcmim]Cl by exchanging the counterion according to a literature method^33^. 1-Thiocyanomethyl-3-methylimidazolium chloride ([C1SCNmim]Cl) was prepared according to a literature method^34^. d_6_-DMSO (99.96%) and d_7_-DMF (99.50%) were purchased from Apollo. LiCl (99%, anhydrous) was purchased from Acros Organics. Deionized water was purified using a Millipore Milli-Q system. All other solvents (anhydrous, >99.9%) were purchased from commercial suppliers and used as received.

### Reaction of [Bcmim]Cl and PbI_2_: synthesis of [Bcmim]_2_Pb_3_Cl_2_I_6_·2DMF

[Bcmim]Cl (18.3 mg, 0.1 mmol) and PbI_2_ (46.1 mg, 0.1 mmol) were mixed in DMF (1.0 ml) and the resulting mixture was heated at 65 °C for 1 h. The solvent was evaporated slowly at room temperature and crystals formed. The crystals were analysed by single-crystal X-ray diffraction (SC-XRD).

### Reaction of [Bcmim]Cl with PbI_2_ and [MA]I or [FA]I: synthesis of [Bcmim]PbI_3_·DMF

[Bcmim]Cl (18.3 mg, 0.1 mmol), MAI (15.9 mg, 0.1 mmol) and PbI_2_ (46.1 mg, 0.1 mmol) were mixed in DMF (1 ml) and the mixture was heated at 65 °C for 1 h to form a yellow solution. The solvent was allowed to evaporate at room temperature and crystals formed. The crystals were analysed by SC-XRD. The reaction of [Bcmim]Cl (18.3 mg, 0.1 mmol), FAI (17.9 mg, 0.1 mmol) and PbI_2_ (46.1 mg, 0.1 mmol) resulted in the same product, as determined by SC-XRD.

### Single-crystal characterization

Single, colourless, prism-shaped crystals of [Bcmim]_4_Pb_3_Cl_2_I_6_·2DMF and [Bcmim]PbI_3_·DMF were obtained and measured. Suitable crystals with dimensions of 0.42 × 0.07 × 0.04 mm^3^ (for [Bcmim]_4_Pb_3_Cl_2_I_6_·2DMF) and 0.40 × 0.05 × 0.04 mm^3^ (for [Bcmim]PbI_3_·DMF) were selected and mounted on a SuperNova, Dual, Cu at home/near, Atlas diffractometer. The crystal was kept at *T* = 140.00(10) K during data collection. The structure was solved with the ShelXT-2018/2 (Sheldrick, 2015) solution program using dual methods and by using Olex2 1.3 (Dolomanov et al., 2009) as the graphical interface. The model was refined with ShelXL 2018/3 (Sheldrick, 2015) using full-matrix least-squares minimization on *F*^2^.

### Fabrication of TiO_2_ compact layer

The patterned FTO substrate (Asahi FTO glass, 12–13 Ω per square) was sequentially cleaned using acetone, isopropanol and deionized water for 15 min by means of ultrasonic cleaning. A 2 M aqueous TiCl_4_ stock solution was first prepared by mixing TiCl_4_ with deionized water at 0 °C and then stored in a refrigerator at 5 °C. The cleaned FTO substrate was soaked in a dilute TiCl_4_ solution (1:20 molar ratio of 2 M aqueous TiCl_4_ stock solution and deionized water) and placed in a sealed glass container. Then the glass container was put in a drying cabinet at 70 °C for 1 h. After cooling, the FTO substrate was rinsed with ethanol and deionized water three times and annealed at 120 °C for 1 h.

### Fabrication of SnO_2_-modified TiO_2_ (SnO_2_@TiO_2_) compact layer

A 2 M SnCl_2_ stock solution in ethanol was first prepared by dissolving SnCl_2_ in ethanol and then stored in the refrigerator at 5 °C. The FTO substrate coated with TiO_2_ nanoparticles was soaked in a dilute SnCl_2_ solution (1:50 molar ratio of 2 M aqueous TiCl_4_ stock solution and cold deionized water) in a glass container. Then the glass container was placed in a drying cabinet at 70 °C for 1 h. After cooling down, the FTO substrate was rinsed with ethanol and deionized water three times. Finally, the substrate was annealed on a hotplate at 180 °C for 1 h.

### Fabrication of PSCs

The PMMA:PCBM solution was prepared by mixing 1 mg of PMMA and 3 mg of PCBM in 1 ml of chlorobenzene. The PMMA:PCBM solution was spin-coated onto the SnO_2_@TiO_2_ layer at 5,000 rpm for 15 s. Subsequently, the substrate was annealed at 100 °C for 10 min.

A pristine precursor solution was prepared by mixing CsCl (0.065 M), MAI (0.065 M), FAI (1.17 M) and PbI_2_ (1.365 M) in a solvent mixture of DMF and DMSO (4:1 v/v). The [Bcmim]Cl-only precursor solution was prepared by mixing CsCl (0.065 M), MAI (0.065 M), FAI (1.17 M), PbI_2_ (1.365 M) and [Bcmim]Cl (0.0078 M) in a solvent mixture of DMF and DMSO (4:1 v/v). The control precursor solution was prepared by mixing CsCl (0.065 M), MAI (0.065 M), FAI (1.17 M), PbI_2_ (1.365 M) and MACl (0.26 M) in a solvent mixture of DMF and DMSO (4:1 v/v). The target precursor solution was prepared by mixing CsCl (0.065 M), MAI (0.065 M), FAI (1.17 M), PbI_2_ (1.365 M), MACl (0.26 M) and [Bcmim]Cl (0.0078 M) in a solvent mixture of DMF and DMSO (4:1 v/v). The solution was then stirred for 3 h at 60 °C. The PPS was spin-coated onto the substrate surface at 1,000 rpm for 10 s, then accelerated to 4,000 rpm for 3 s and maintained at this speed for 20 s. The substrate was placed in a home-built rapid vacuum-drying apparatus, as previously reported^35^. After pumping for 20 s, a brown, semi-transparent perovskite film with a mirror-like surface was obtained. The fresh perovskite layer was annealed at 100 °C for 1 h and then at 150 °C for 10 min. Afterwards, 60 μl of PEAI isopropanol solution (5 mg ml^−1^) was deposited on the perovskite film by spin-coating at 5,000 rpm for 30 s.

A hole-transport layer was deposited on the perovskite film by spin-coating doped Spiro-OMeTAD solution at 3,000 rpm for 30 s. The doped Spiro-OMeTAD solution was prepared by dissolving Spiro-OMeTAD (32 mg), PEDOT:complex HTL Solar 3 (0.5 ml) and tris(pentafluorophenyl)borane (8 mg) in chlorobenzene (1 ml). A 5-nm-thick layer of MoO_3_ was thermally evaporated onto the Spiro-OMeTAD layer. Subsequently, a 100-nm-thick film of indium tin oxide (ITO) was sputtered onto the MoO_3_ layer. Finally, a back electrode consisting of an approximately 50-nm-thick layer of gold was evaporated.

### Fabrication of PSMs based on spin-coating method

PSMs with eight subcells connected in series were fabricated on FTO glass with a size of 6.5 × 7.0 cm^2^. The series interconnection of the module was realized by P1, P2 and P3 lines, which were patterned using a laser scribing system with a wavelength of 1,064 nm and a power of 20 W (Trotec). The FTO substrate was pre-patterned for P1 (40 μm wide) at 60% laser power and a speed of 300 mm s^−1^ with a frequency of 65 kHz and pulse width of 120 ns. The subsequent processes for the preparation of SnO_2_@TiO_2_ substrates, PMMA:PCBM layer, perovskite layer, PEAI layer and doped Spiro-OMeTAD layer are the same as the small-area-device procedures. The P2 lines (100 μm wide) were patterned with an average laser power of 15% at a speed of 1,000 mm s^−1^ and frequency of 65 kHz for a pulse duration of 120 ns. After MoO_3_/ITO/Au layers were sequentially deposited, the P3 lines (40 μm wide) were patterned with an average laser power of 18% at a speed of 1,000 mm s^−1^ and frequency of 65 kHz for a pulse duration of 120 ns. The distance between the P1 and P3 lines is about 280 μm and the geometric fill factor is around 95.74%.

### Fabrication of PSMs using blade-coating

Laser etching and cleaning of FTO glass, as well as the preparation of the compact SnO_2_@TiO_2_ electron-transport layer by chemical-bath deposition, were conducted as previously described. Subsequently, the PMMA:PCBM solution was blade-coated onto the SnO_2_@TiO_2_/FTO substrate and the substrate was annealed at 100 °C for 10 min. Next, a MACl-based precursor solution was prepared by mixing CsCl (0.04 M), MAI (0.04 M), FAI (0.72 M), PbI_2_ (0.84 M) and MACl (0.16 M) in DMF/DMSO (4:1 v/v). A MACl/[Bcmim]Cl-based precursor solution was prepared by mixing CsCl (0.04 M), MAI (0.04 M), FAI (0.72 M), PbI_2_ (0.84 M), MACl (0.16 M) and [Bcmim]Cl (0.0048 M) in DMF/DMSO (4:1 v/v). The perovskite solution was blade-coated onto the PMMA:PCBM/SnO_2_@TiO_2_/FTO substrate. The as-coated perovskite liquid layer was dried using a gas-pump method, then annealed at 100 °C for 1 h and at 150 °C for 10 min. A PEAI solution (7:3 v/v of isopropanol:chlorobenzene) was blade-coated onto the perovskite film. A N_2_ air knife was used to facilitate drying throughout the coating process. Subsequently, the Spiro-OMeTAD solution was blade-coated onto the perovskite film. The N_2_ air knife was used to assist drying during the coating process. The P2 lines, MoO_3_/ITO/Au layers and P3 lines were conducted as previously described.

### Module encapsulation

The modules were encapsulated by glass–glass encapsulation technology combined with an edge seal (UV Curing Sealant, ThreeBond 3035B) of the module under UV light illumination (LED flood lamp, DELOLUX 20). First, the edge of the device was laser-cleaned. An indium solder was laid on the FTO and Au electrodes on the edge of the 65 × 70-mm^2^ substrate. Then, a glass piece (60 × 65 mm^2^) was placed on top of the Au layer of the device. A light-curing sealant was deposited on the edges of the glass to fully cover the gap between the top glass piece and the module. Finally, cross-linking of the sealant was induced by a UV light at 25% of maximum power for 120 s in a glovebox.

### Film characterization

#### XRD and 2D GIWAXS

The phase composition of the films was characterized by XRD with a Bruker D8 Advance diffractometer equipped with a Cu Kα radiation source (*λ* = 1.5418 Å). GIWAXS patterns represented in reciprocal lattice space were conducted at beamline BL46XU of SPring-8. The samples were irradiated with X-ray energy of 12.39 keV (*λ* = 1 Å) at a fixed incident angle on the order of 2.0° using a Huber diffractometer. The GIWAXS patterns were recorded with a 2D image detector (Pilatus 300 K).

#### Observation of the crystallization process

To record the crystallization process of the perovskite films, the PPS was first spin-coated onto the FTO substrate. Wet perovskite precursor films were then mounted on an in situ heating holder at 100 °C and observed with a microscope (DM2500P, Leica) equipped with a hot stage (LTSE420, Linkam) and a camera (MicroPublisher 5.0 RTV, QImaging). PPSs with 0, 0.2, 0.6, 1.0 and 1.4 mol% of [Bcmim]Cl (with respect to 1.0 M PbI_2_) were studied.

#### DLS

The characterization of DLS was conducted using a Zetasizer Nano ZS particle size analyser from Malvern Instruments Ltd. The concentration of the PPS used for the DLS measurement was equivalent to that used in the device fabrication.

#### Morphological characterization

Top-view and cross-sectional morphologies of the perovskite films and devices were characterized using a scanning electron microscope (FEI Sirion 200) with a voltage of 5 kV and a current of 0.1 nA.

#### UV–Vis absorption and X-ray photoelectron spectroscopy

Absorption spectra of the perovskite films were collected using a UV–Vis absorption spectrophotometer (PerkinElmer Lambda 950S). X-ray photoelectron spectroscopy measurements were conducted using a VersaProbe II (Physical Electronics Inc.) equipped with a monochromator and Al Kα source (1,486.6 eV). The spectrum was referenced using the C–C-bound component of adventitious carbon.

#### PL and PLQY characterization

Steady-state PL mapping was carried out with a laser confocal Raman spectrometer (Princeton Instruments, Acton Standard Series SP-2558), a digital charge-coupled device (PIXIS: 100B_eXcelon) and a 488-nm laser (PicoQuant LDH-P-C-485, 0.4 mW with a 1% optical density filter), using a home-built confocal microscope on an area of 1 × 1 mm^2^. TRPL spectra of films were obtained using a time-correlated single-photon counting system (Nanofinder 30) with an excitation wavelength of 480 nm. PLQY spectra were acquired following a three-step procedure^36^, using an FLS1000 Photoluminescence Spectrometer, coupled with an integrating sphere (N-M01, Edinburgh Instruments). Samples were excited at a wavelength of 635 ± 10 nm and emission was recorded between 700 and 870 nm.

#### Device characterization

All devices were measured using an Oriel solar simulator (450 W xenon, AAA class) equipped with a Keithley 2400 source meter at a light intensity of 100 mA cm^−2^, calibrated using a Si reference solar cell (KG3, Newport). A black metal mask (0.06 cm^2^) was applied to define the testing area of PSCs. PSCs were measured in forward and reverse scan modes with a bias voltage from 0 to 1.2 V at a scanning step of 10 mV and a delay time of 500 ms. PSMs were measured in forward and reverse scan modes with a bias voltage from 0 to 9.5 V at a scanning step of 50 mV and a delay time of 200 ms with a metal mask (27.22 cm^2^). External quantum efficiency spectra were measured using an IQE-200B (Oriel) system without bias light.

#### Operational stability test of modules

*I*–*V* curves were measured using an electronic system equipped with a 22-bit delta-sigma analogue-to-digital converter. A reference Si photodiode was placed next to the modules to record the light intensity. The encapsulated modules were maintained at the MPP using an MPP tracking algorithm under simulated solar light (AM 1.5 G, 100 mW cm^−2^) according to the ISOS-L-1 protocol^30^. The encapsulated modules were placed on a hotplate set to 65 °C to measure their stability using the ISOS-L-2 protocol^30^.

#### Thermal stability tests

The perovskite films were annealed at 60 ± 5 °C on a hotplate in a N_2_ glovebox to explore their thermal stability. Changes were tracked using XRD.

#### HIM-SIMS characterization

The surface morphologies of perovskite films were initially characterized through images obtained in secondary electrons mode. The images were collected by scanning the surface using a helium beam (20 keV, 1 pA) from a helium ion microscope (Zeiss). This procedure aligns with method described in the literature^37^ and produces images similar to those from SEM. The helium ion microscope was coupled with a secondary ion mass spectrometer and SIMS images were acquired using a high-energy neon (Ne^+^) primary ion beam (20 keV, 3 pA). The Ne^+^ beam was sourced from the same column as the helium ion beam but is more efficient at sputtering. Consequently, the Ne^+^ beam results in a stronger signal, making it more suitable for SIMS analysis^37^. Sweeping the Ne^+^ beam across the surface induces the emission of secondary ions, which were collected, mass-filtered and counted using the SIMS instrument. HIM-SIMS, with a sub-15-nm lateral resolution and high chemical sensitivity, facilitates the identification and differentiation of submicron grain compositions^37^. SIMS images of the perovskite films were collected in a 512 × 512-pixel matrix with a 10 × 10-µm^2^ (19.5 nm per pixel) field of view. To assess the phase distribution in the initial perovskite films, we performed compositional analysis on films fabricated by drying with a gas pump for 120 s to minimize residual solvent present before storage at room temperature. For the phase distribution in the final perovskite films, the films were annealed in two steps, first at 100 °C for 60 min and second at 150 °C for 10 min.

Depth-dependent chemical composition variations were studied using ToF-SIMS (Münster) with bismuth (Bi^+^) as the analytical primary ion source (25 keV, 0.37 pA). A low-energy Cs^+^ ion beam (2 keV, 20 nA) was used to sputter the sample between two successive raster scans with the Bi^+^ beam. The depth profile was collected by alternating sputtering and acquisition analysis steps on representative areas of 500 × 500 µm^2^ and 200 × 200 µm^2^, respectively. The analysis time was used as a proxy for depth in the profile and was analytically transformed into a depth estimate. The intensity of secondary ions summed over the analysed area was represented as a data point in the profile and plotted against analysis time/depth. Ions were monitored either as ion M^+^ (for example, Pb^+^, [Bcmim]^+^) or their MCs^+^ caesium cluster (for example, CsI^+^, CsCl^+^).

#### TRMC measurements

Time-resolved microwave conductivity (TRMC) measurements were used to determine the mobility of photogenerated charge carriers. Perovskite films fabricated on sapphire substrates were placed in a microwave resonator with a resonance frequency of 9 GHz. The charge carriers, generated with the second harmonic of a Nd:YAG laser (532 nm), led to an increase of the conductance *G* of the perovskite film. The normalized change of reflected microwave power $\frac{\Delta P}{P}$ from the resonator, as measured, related to the change in conductance Δ*G* between a dark and illuminated sample and a sensitivity factor *K*, obtained by further resonance measurements, according to equation (1). Finally, to extract the sum of the electron and hole mobility Σ*μ* according to equation (2), the maximum change of the conductance Δ*G*_Max_, the absorption *F*_A_ of the perovskite film, the illumination intensity *I* and the ratio of the resonator dimensions *β* are needed. *ϕ* describes a free carrier generation yield, which is close to unity for low-excitation fluences and *e* is the elementary charge. All measurements were carried out in air at room temperature.

1 $$\frac{\Delta P}{P}=K\cdot \Delta G$$

2 $$\phi \Sigma \mu =\frac{{\Delta G}_{{\rm{Max}}}}{e\beta I{F}_{{\rm{A}}}}$$

A detailed description of the setup and measurement method can be found in ref. ^38^.

#### NMR spectroscopy

NMR spectra were acquired on a Bruker 400 MHz spectrometer (9.4 T) equipped with a console AVIII HD and a 5-mm liquid-state BBO Z-gradient field three-channel (^1^H/^2^H/BBF) probe head. Experiments were performed at room temperature while locking to the deuterium signal of the d_6_-DMSO. ^1^H and ^13^C chemical shifts were referenced to Si(CH_3_)_4_ (δ(^1^H, ^13^C) = 0 ppm) using the signals of the residual protons and of the ^13^C of the deuterated solvent (δ^1^H = 2.50 ppm, δ^13^C = 39.52 ppm (d_6_-DMSO)) as secondary reference. ^11^B and ^31^P chemical shifts were indirectly referenced with the ^1^H signals of the residual protons of the deuterated solvent using the Ξ‐scale (Ξ = 32.083974 MHz and 40.480747 MHz, respectively) with 15% BF_3_OEt_2_ in CDCl_3_ (δ(^11^B) = 0 ppm) or 85% H_3_PO_4_ (δ(^31^P) = 0 ppm) as secondary references. 1D ^1^H, ^13^C, ^11^B and ^31^P spectra were acquired using the standard pulse sequences from the Bruker library. 2D COSY, HSQC and HMBC NMR spectra were acquired using the standard pulse sequences from the Bruker library. NMR spectra were processed with MestreNova 14.2.1 (Mestrelab Research S.L.) and TopSpin 4.2.0 (Bruker).

#### NMR spectroscopic assessment of [Bcmim]Cl and MACl interactions

The interaction between [Bcmim]Cl and MACl in solution was assessed by ^1^H NMR spectroscopy. To a stock solution of MACl (0.3 M in d_6_-DMSO) in a 5-mm NMR tube, different volumes of [Bcmim]Cl (0.3 M in d_6_-DMSO) were added to obtain [Bcmim]Cl:MACl molar ratios from 0 to 100%. The final volume was adjusted to 0.6 ml with d^6^-DMSO. Control experiments were performed using the same methodology for [Dmim]Cl, [Bmim]Cl and [Cmmim]Cl. The effect of the anion in MAX was also studied by replacing MACl by MAI. The concentration dependence of [Bcmim]Cl peak shifts was studied by adding 5, 10, 15, 20 or 25 μl of a 0.15 M [Bcmim]Cl stock solution (0.0138 g, 0.0756 mmol in 0.5 ml d_6_-DMSO) to a 5-mm NMR tube. The final volume inside the tube was adjusted to 0.5 ml with d_6_-DMSO, resulting in 0.0015, 0.0030, 0.0045, 0.0060 and 0.0075 M of [Bcmim]Cl (equivalent to the concentrations in the 1, 2, 3, 4 and 5 mol% samples in the [Bcmim]Cl and MACl for interaction study).

#### Hydrogen-bond-accepting ability

The hydrogen-bond-accepting ability of the selected ionic liquids ([Bcmim]Cl and [Bcmim]TFSI) was compared by studying the changes in the ^31^P NMR chemical shift using phenylphosphinic acid (PPA) as a hydrogen-bond-accepting probe following a previously reported method^25^. The ionic liquid (3.0 eq., 0.0275 mmol) was dissolved in 0.4 ml of dry DMSO and transferred to an oven-dried 5-mm NMR tube and sonicated to ensure complete dissolution. Then, 0.15 ml (1.0 eq., 0.0092 mmol) of a stock solution of PPA (0.0611 M in d_6_-DMSO) was added to the tube. The ^31^P NMR spectrum of each sample was recorded at room temperature with 16 scans and compared with a 0.0166 M external standard of PPA (0.15 ml PPA stock solution in d_6_-DMSO + 0.4 ml of DMSO) to determine the difference in the chemical shift.

#### In operando time-dependent and variable-temperature NMR spectroscopy experiments

Time-dependent and variable-temperature NMR spectra were acquired on a Bruker 400 MHz spectrometer (9.4 T) equipped with a console AVIII HD and a BBO Z-gradient field three-channel (^1^H/^2^H/BB) liquid-state probe head for 10-mm NMR tubes. Custom-made (built in-house according to a literature protocol)^39,40^ high-pressure 10-mm sapphire NMR tubes (with a length of 130 mm and an internal diameter of 8 mm) were used. Experiments were performed at variable temperatures in non-spinning mode while locking to the deuterium signal of d_6_-DMSO. ^1^H and ^13^C NMR chemical shifts were referenced to Si(CH_3_)_4_ (δ(^1^H, ^13^C) = 0 ppm) using the signals of the residual protons and of the ^13^C of the deuterated solvent (δ^1^H = 2.50 ppm, δ^13^C = 39.51 ppm (d_6_-DMSO)) as secondary reference. ^11^B and ^207^Pb chemical shifts were indirectly referenced with the ^1^H signals of the residual protons of the deuterated solvent using the Ξ‐scale (Ξ = 32.083974 MHz and 20.920599 MHz, respectively) with 15% BF_3_·OEt_2_ in CDCl_3_ (δ(^11^B) = 0 ppm) or Me_4_Pb + 5% C_6_D_6_ (δ(^207^Pb) = 0 ppm) as secondary references. ^1^H, ^13^C, ^11^B and ^207^Pb spectra were acquired using the standard pulse sequences from the Bruker library. 2D COSY, HSQC and HMBC spectra were acquired using the standard pulse sequences from the Bruker library, accumulating 16 scans and 128 points in the indirect dimension for the COSY spectra and 16 scans and 512 points in the indirect dimension for the HSQC and HMBC spectra.

Lead-perovskite film precursor solutions were prepared by mixing CsCl (0.065 M), MAI (0.065 M), FAI (1.17 M), PbI_2_ (1.365 M) and MACl (0.26 M) in 2 ml of d_7_-DMF and d_6_-DMSO (4:1 v/v) in the presence and absence (control) of [Bcmim]Cl (0.6 mol%, 0.78 mM). The mixtures were sonicated to ensure complete dissolution and transferred to the 10-mm sapphire NMR tubes. The stability of the solutions was analysed using in operando NMR spectroscopy. Variable-temperature experiments were performed between 25 and 100 °C with 5 °C increments. The samples were allowed to equilibrate to the corresponding temperature for 600 s before acquiring the spectra. 1D spectra were acquired with one scan for ^1^H NMR spectra, 16 scans for ^13^C NMR spectra and 32 scans for ^207^Pb NMR spectra. The 90° pulse and the recovery delay for ^1^H, ^13^C and ^207^Pb NMR spectra were 25 μs and 600 s, 15 μs and 3.0 s and 15 μs and 2.0 s, respectively. ^207^Pb NMR spectra were recorded between −3,500 and +2,000 ppm with 1,000-ppm-width subwindows, retuning the probe at each step. Time-dependent experiments were obtained using a pseudo-2D sequence consisting of ^1^H NMR spectra recorded every 15 min for 24 h. Time-dependent experiments were performed at 25 and 60 °C. The samples were allowed to equilibrate to the corresponding temperature for 600 s before acquiring the spectra. 96 points in the indirect dimension were recorded, each consisting of a one-pulse sequence with 90° flip angle of 25 μs. EXSY spectra (a phase-sensitive 2D homonuclear correlation through dipolar coupling experiment that can detect chemical exchange) were acquired using a modified standard pulse sequence from the Bruker library. 1,024 points in the indirect dimension with an increment of 208.27 μs were recorded, each consisting of 16 scans and 2,048 data points (AQ = 0.21 s) using purge pulses before D1. A mixing time of 0.3 s was set as a simple delay and a recycle delay (D1) of 2.0 s was used.

#### pH measurements

The studied ionic liquids ([Dmim]Cl, [Bmim]Cl, [Cmmim]Cl, [Bcmim]TFSI and [Bcmim]Cl) (0.0075 mmol, equivalent to 5 mol% of 0.15 M) were dissolved in 10.0 ml deionized H_2_O and sonicated to ensure complete dissolution. The pH was then measured at room temperature on a Metrohm 780 pH meter and corrected to 25 °C.

#### Computational details

All simulations were performed using DFT, the Perdew–Burke–Ernzerhof density functional with Grimme’s D3 dispersion^41^ correction. The wavefunction was expanded in the localized DZVP-MOLOPT basis set^42^, an auxiliary plane-wave basis set with the 600 Ry kinetic energy cutoff was used for the expansion of electron density and the core region was described with norm-conserving Goedecker–Teter–Hutter^43^ pseudopotentials. The Brillouin zone was sampled at the Γ-point. The crystal structure of bulk perovskite Cs_0.05_MA_0.05_FA_0.90_PbI_3_ was fully relaxed, including both atomic positions and cell parameters. The atomic positions of surface structures, including those having adsorbed species, were also fully optimized. To simulate the deposition of an ionic liquid on the perovskite surfaces, Born–Oppenheimer molecular dynamics runs were performed at a constant temperature of 300 K for several picoseconds with a 1-fs time step using a velocity-rescaling algorithm. All simulations were accomplished with the CP2K code^44^. The structures were visualized using VESTA^45^ and VMD^46^ software.

## Online content

Any methods, additional references, Nature Portfolio reporting summaries, source data, extended data, supplementary information, acknowledgements, peer review information; details of author contributions and competing interests; and statements of data and code availability are available at 10.1038/s41586-024-07228-z.

### Supplementary information

Supplementary Information This file contains Supplementary Notes 1 and 2, Supplementary Figs. 1–33, Supplementary Tables 1–5 and Supplementary References.

## Data Availability

The data that support the findings of this study are available from the corresponding authors on reasonable request.
