# Supplementary material for: Dopant-additive synergism enhances perovskite solar modules
Source: Nature. 2024 Mar 4;628(8007):299–305. doi: 10.1038/s41586-024-07228-z (PMC11006611; doi:10.1038/s41586-024-07228-z)
Supplement: Supplementary file 1 — This file contains Supplementary Notes 1 and 2, Supplementary Figs. 1–33, Supplementary Tables 1–5 and Supplementary References. [file 41586_2024_7228_MOESM1_ESM.pdf]

nature portfolio

https://doi.org/10.1038/s41586-024-07228-z

Supplementary information

Dopant-additive synergism enhances perovskite solar modules

In the format provided by the authors and unedited

Supplementary Information for:

Dopant-additive synergism enhances perovskite solar modules

Bin Ding[1,†], Yong Ding[1,2,†,*], Jun Peng[3,†], Jan Romano-deGea[1,†], Lindsey E. K. Frederiksen[1,†], Hiroyuki Kanda[1], Olga A. Syzgantseva[4], Maria A. Syzgantseva[4], Jean-Nicolas Audinot[5], Jerome Bour[5], Song Zhang[6], Tom Wirtz[5], Zhaofu Fei[1,*], Patrick Dörflinger[7], Naoyuki Shibayama[8], Yunjuan Niu[9], Sixia Hu[10], Shunlin Zhang[1], Farzaneh Fadaei Tirani[1], Yan Liu[11], Guan-Jun Yang[11], Keith Brooks[1], Linhua Hu[9], Sachin Kinge[12], Vladimir Dyakonov[7], Xiaohong Zhang[3,*], Songyuan Dai[2], Paul J. Dyson[1,*] and Mohammad Khaja Nazeeruddin[1,*]

[1]Institute of Chemical Sciences and Engineering, École Polytechnique Fédérale de Lausanne (EPFL), 1015 Lausanne, Switzerland.

[2]State Key Laboratory of Alternate Electrical Power System with Renewable Energy Sources, North China Electric Power University, Beijing 102206, P. R. China.

[3]Institute of Functional Nano & Soft Materials (FUNSOM), Jiangsu Key Laboratory of Advanced Negative Carbon Technologies, Joint International Research Laboratory of Carbon-Based Functional Materials and Devices, Soochow University, Suzhou, Jiangsu 215123, P. R. China.

[4]Department of Chemistry, Lomonosov Moscow State University, Moscow 119991, Russia.

[5]Advanced Instrumentation for Nano-Analytics (AINA), Materials Research and Technology Department, Luxembourg Institute of Science and Technology (LIST); L-4422 Belvaux, Luxembourg.

[6]State Key Laboratory of Magnetic Resonance and Atomic and Molecular Physics, Innovation Academy for Precision Measurement Science and Technology, Chinese Academy of Sciences, Wuhan 430071, P. R. China.

[7]Institute of Physics, Julius Maximilian University of Würzburg, 97074 Würzburg, Germany.

[8]Faculty of Biomedical Engineering, Graduate School of Engineering, Toin University of Yokohama, 1614 Kurogane-cho, Aoba, Yokohama, Kanagawa 225-8503, Japan.

[9]Key Laboratory of Photovoltaic and Energy Conservation Materials, CAS, Institute of Solid-State Physics, Hefei Institutes of Physical Science, Chinese Academy of Sciences, Hefei, Anhui,

230031, P. R. China.

[10]Materials Characterization and Preparation Center, Southern University of Science and Technology, Shenzhen 518055, P.R. China.

[11]State Key Laboratory for Mechanical Behavior of Materials, School of Materials Science and Engineering, Xi'an Jiaotong University, 28 West Xianning Road, Xi'an, Shaanxi, 710049, P. R. China.

[12]Toyota Motor Corporation, Toyota Motor Technical Centre, Advanced Technology Div., Hoge Wei 33, B-1930 Zaventem, Belgium.

[†]These authors contributed equally: Bin Ding, Yong Ding, Jun Peng, Jan Romano-deGea and Lindsey E. K. Frederiksen.

[*]E-mail: dingy@ncepu.edu.cn; zhaofu.fei@epfl.ch; xiaohong_zhang@suda.edu.cn; paul.dyson@epfl.ch; mdkhaja.nazeeruddin@epfl.ch.

This PDF file includes:

Supplementary Notes 1 to 2

Figures S1 to S33

Tables S1 to S5

References 1-14

Content

Supplementary Notes 5
Supplementary Note 1 5
Supplementary Note 2 6
Supplementary Figures 7
Supplementary Fig. S1 7
Supplementary Fig. S2 8
Supplementary Fig. S3 9
Supplementary Fig. S4 10
Supplementary Fig. S5 11
Supplementary Fig. S6 12
Supplementary Fig. S7 13
Supplementary Fig. S8 14
Supplementary Fig. S9 15
Supplementary Fig. S10 16
Supplementary Fig. S11 17
Supplementary Fig. S12 18
Supplementary Fig. S13 19
Supplementary Fig. S14 20
Supplementary Fig. S15 21
Supplementary Fig. S16 22
Supplementary Fig. S17 23
Supplementary Fig. S18 24
Supplementary Fig. S19 26
Supplementary Fig. S20 27
Supplementary Fig. S21 29
Supplementary Fig. S22 30
Supplementary Fig. S23 31
Supplementary Fig. S24 32

Supplementary Fig. S25 33
Supplementary Fig. S26 34
Supplementary Fig. S27 35
Supplementary Fig. S28 36
Supplementary Fig. S29 37
Supplementary Fig. S30 38
Supplementary Fig. S31 39
Supplementary Fig. S32 40
Supplementary Fig. S33 42
Supplementary Tables 43
Supplementary Table S1 43
Supplementary Table S2 45
Supplementary Table S3 46
Supplementary Table S4 47
Supplementary Table S5 48
References 49

Supplementary Notes

Supplementary Note 1

Other control experiments were required to indirectly rule out possible alternative explanations for the observed effects. Therefore, ^{1}H NMR spectra of 0.15 M MACl solutions (d_6-DMSO) containing 5 mol% LiCl, HCl, or trifluoroacetic acid (TFA) were measured both with and without 5 mol% [Bcmim]Cl. LiCl was used to probe the effect of solvated Cl^- (an excellent HBA) and HCl represents the acid formed by deprotonation of [Bcmim]Cl. To probe the influence of pH decoupled from Cl^- interference, TFA was used as a source of H^+ with a non-coordinating counter-anion. The results indicate that the Cl^- of [Bcmim]Cl is responsible for the downfield shift of the MA^+ NH peak, but that an interaction, independent of solution pH, between $[Bcmim]^+$ and MA^+, related to inherent acidity of $[Bcmim]^+$, is responsible for the peak broadening.

Effect of solvated Cl^- ions: A 0.13 M stock solution of LiCl was prepared by dissolving LiCl (0.0011 g, 0.026 mmol) in d_6-DMSO (0.2 mL). MACl (0.0058 g, 0.086 mmol) was combined with 25 μL of LiCl stock in the presence and absence (control) of [Bcmim]Cl. After dilution, the final concentrations were 0.15 M MACl, 0.0065 M LiCl (5 mol%), and 0.0076 M [Bcmim]Cl (5 mol%).

Effect of acidity (solvated H^+ ions): MACl (0.0051 g, 0.075 mmol) was combined with 0.3 μL TFA. After dilution, the final concentrations were 0.15 M MACl and 0.0078 M TFA (5 mol%).

Effect of acidity (solvated H^+ ions) and solvated Cl^- ions: MACl (0.0049 g, 0.073 mmol) was combined with 1.0 μL HCl (4.0 M in 1,4-dioxane in the presence and absence (control) of [Bcmim]Cl. After dilution, the final concentrations were 0.15 M MACl, 0.0075 M HCl (5 mol%) and 0.0077 M [Bcmim]Cl (5 mol%).

Supplementary Note 2

To assess the impact of [Bcmim]Cl on the perovskite crystallisation process, two crystal plane orientations were considered for construction of the surface models: the (110) plane, observed in the GIWAXS experiment, and the (001) surface perpendicular to it. In a cubic perovskite, the (220) and (110) planes are equivalent. The stoichiometric (110) surface of the cubic perovskite has a mixed termination formed by both PbI_2 and (FA, MA, Cs)I species. Therefore, all types of sites are present on the surface, including undercoordinated Pb^{2+} sites, which remove I^- from Pb-I dangling bonds to create charge neutrality on the surface. In contrast, the neutral (001) surface is either PbI_2 or (FA, MA, Cs)I terminated. The PbI_2 termination does not have Pb-I dangling bonds but instead has highly-exposed undercoordinated Pb^{2+} sites. Thus, the Pb^{2+} sites on the (110) surface is partially occupied by I^-, while on the PbI_2-terminated (001) surface they are fully exposed.

The Pb^{2+} sites of the perovskite surfaces can induce binding of [Bcmim]Cl to the surface through the ionic liquid's nitrile functional groups. In addition, weak van der Waals interactions are responsible for the attraction of $[Bcmim]^+$ to the perovskite surface. For both types of (001) surface termination, [Bcmim]Cl tends to adopt planar configurations upon structural optimisation with the nitrile functional groups oriented in-plane with the imidazole ring. It should be stressed that this planar configuration is also observed for the (FA, MA, Cs)I surface terminations. Thus, [Bcmim]Cl tends to passivate the (001) surface, substantially covering it, and thus prevents further growth in the (001) direction. In addition, [Bcmim]Cl can participate in the passivation of grain boundaries terminated by (110) facets, since the two methylene nitrile moieties of one $[Bcmim]^+$ cation can connect two corresponding (110) facets.

This hypothesis is supported by the computed adsorption energy values of neutral-adduct complexes (cation and anion) on the perovskite surfaces according to Equation (1), where E_{adduct} is the energy of the neutral adduct complex in the gas phase.

$$E_{\mathrm{ads}} = E_{\mathrm{surf+adduct}} - E_{\mathrm{surf}} - E_{\mathrm{adduct}} \quad (1)$$

The adsorption of [Bcmim]Cl is more energetically favourable on the (110) surface than on the (001) one. Therefore, [Bcmim]Cl adopts an out-of-plane configuration on the (110) surface, binding to it tighter than to the (001) surface and favouring crystallisation in the (001) direction.

Supplementary Figures

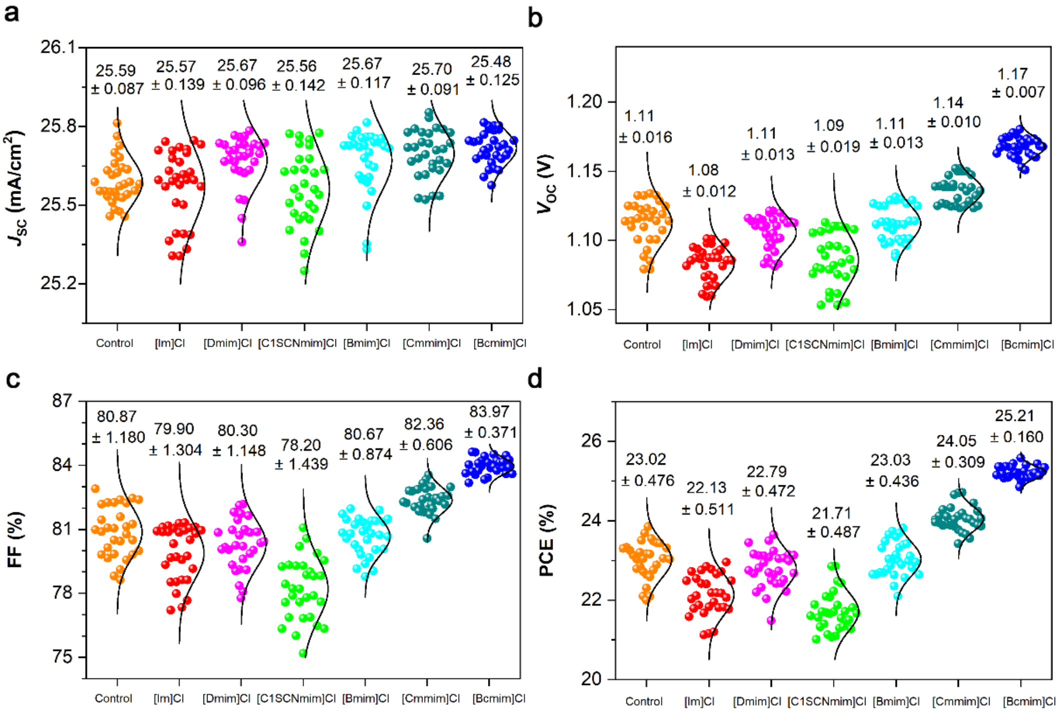

Supplementary Fig. S1 | Statistical distribution of the photovoltaic parameters for PSCs with the combination of MACl and [Im]Cl, [Dmim]Cl, [C1SCNmim]Cl, [Bmim]Cl, [Cmmim]Cl or [Bcmim]Cl. (**a**) Short-circuit current density (J_{SC}), (**b**) open-circuit voltage (V_{OC}), (**c**) fill factor (FF), and (**d**) power conversion efficiency (PCE). Note that the concentration of MACl is 20 mol%, and the concentration of ionic liquids is 0.6 mol%.

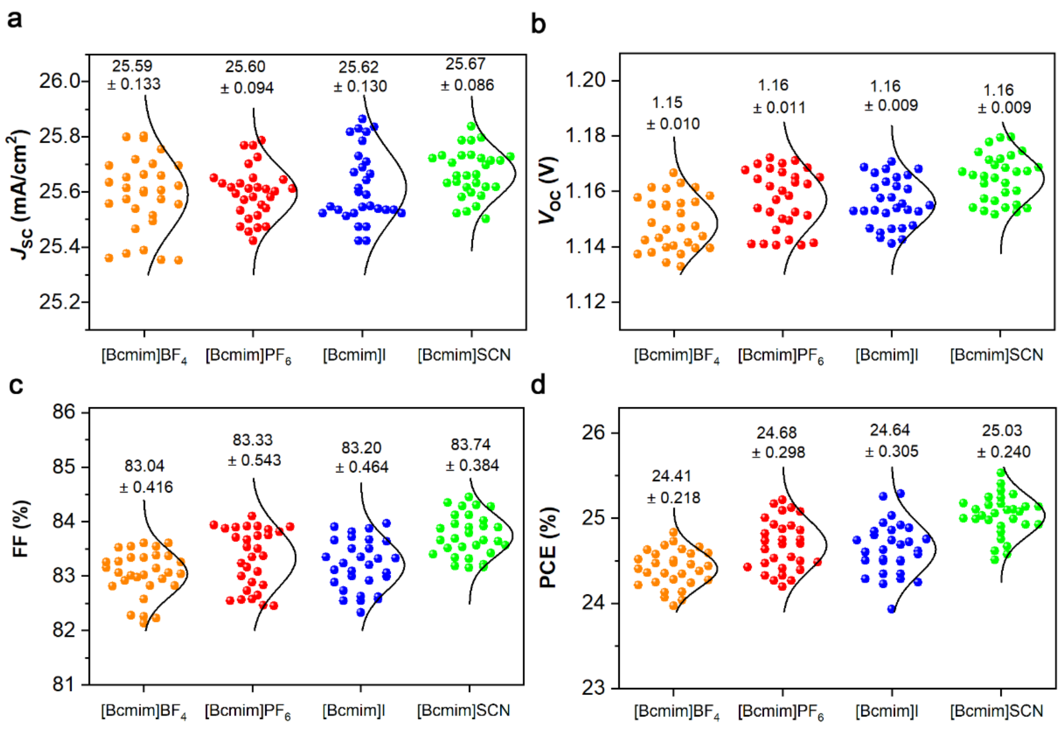

Supplementary Fig. S2 | Statistical distribution of the photovoltaic parameters of PSCs with the combination of MACl and [Bcmim]BF_4, [Bcmim]PF_6, [Bcmim]I or [Bcmim]SCN. (**a**) J_{SC}, (**b**) V_{OC}, (**c**) FF, and (**d**) PCE. Note that the concentration of MACl is 20 mol%, and the concentration of ionic liquids is 0.6 mol%.

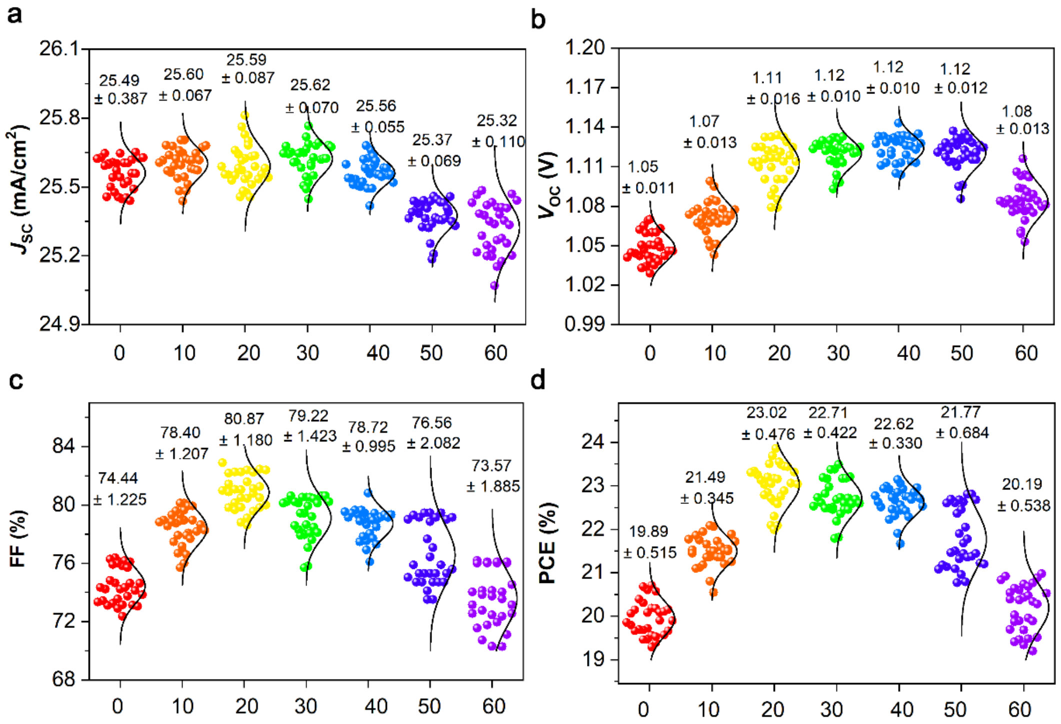

Supplementary Fig. S3 | Statistical distributions of photovoltaic parameters as a function of MACl concentrations from 0 to 60 mol%. (**a**) J_{SC}, (**b**) V_{OC}, (**c**) FF, and (**d**) PCE.

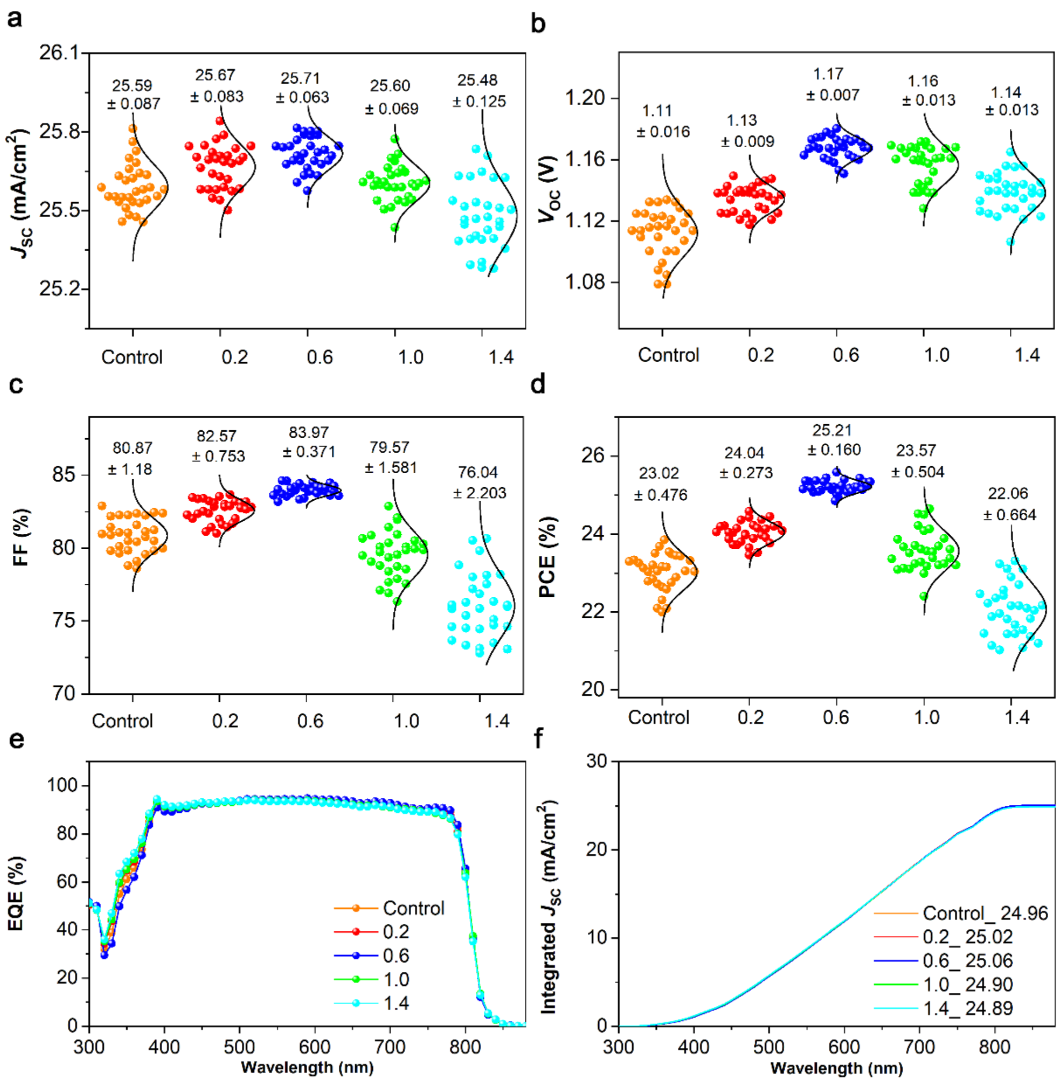

Supplementary Fig. S4 | Statistical distributions of photovoltaic parameters as a function of [Bcmim]Cl concentrations from 0 to 1.4 mol%, while maintaining the MACl concentration constant at 20 mol%. (**a**) J_{SC}, (**b**) V_{OC}, (**c**) FF, and (**d**) PCE. (**e**) The typical EQE spectra and (**f**) the corresponding integrated J_{SC} curve.

a

1 Standard Test Condition (STC)：Total Irradiance：1000 W/m^2

Temperature：25.0 ℃

Spectral Distribution：AM1.5G

2 Measurement Data and I-V/P-V Curves under STC

Forward Scan

I_{sc} (mA)	V_{oc} (V)	I_{MPP} (mA)	V_{MPP} (V)	P_{MPP} (mW)	FF (%)	η (%)
83.98	9.291	77.37	7.459	577.1	73.96	21.20

Reverse Scan

I_{sc} (mA)	V_{oc} (V)	I_{MPP} (mA)	V_{MPP} (V)	P_{MPP} (mW)	FF (%)	η (%)
83.96	9.402	79.61	7.965	634.1	80.33	23.30

Mismatch Factor: 0.9908

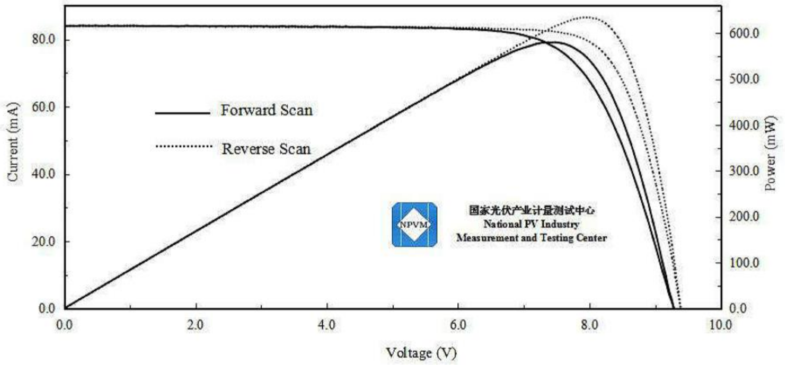

b

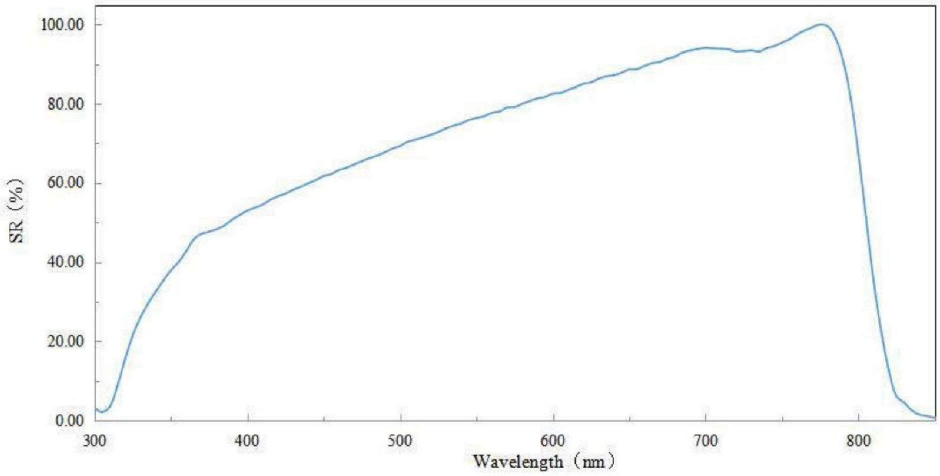

Supplementary Fig. S5 | Certificate of the target PSM. (**a**) *I-V* curve of the certified PSM with average efficiencies of 23.30% under reverse scan and 21.20% under forward scan from three consecutive tests on an aperture area of 27.22 cm^2 from the Chinese National PV Industry Measurement and Testing Centre (NPVM). (**b**) The relative spectral responsivity curve of the certified PSM.

Measurement Data and Curves for MPPT under STC

η (%)	22.50
P_{MPP} (mW)	612.5
I_{MPP} (mA)	79.55
V_{MPP} (V)	7.700

Note: Measurement data for MPPT under STC in the above table was the mean value acquired during 300 sec.

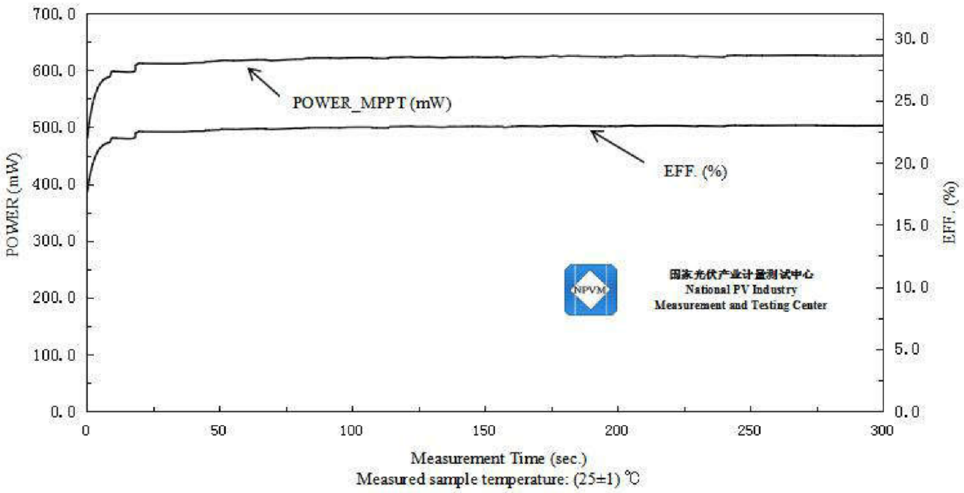

Figure 2. Measurement curves of the measured sample for MPPT

Supplementary Fig. S6 | Steady-state power output performance under 300- second maximum power point tracking of the certified PSM with an average efficiency of 22.50% and final stabilised efficiency of 22.97% from NPVM.

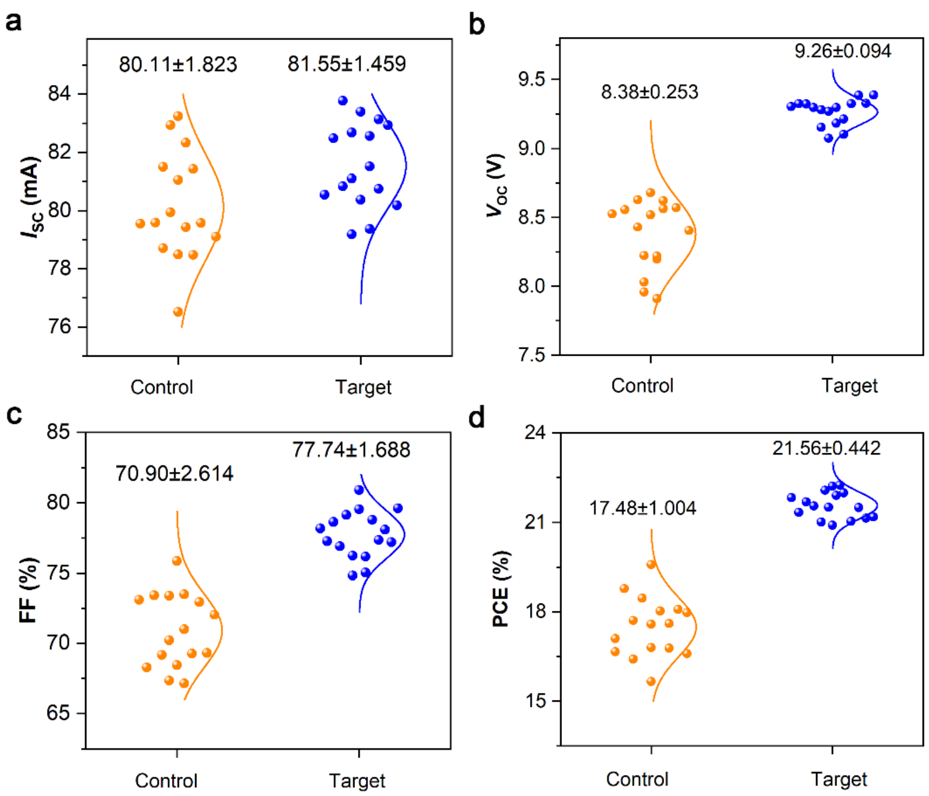

Supplementary Fig. S7 | Statistical distributions of photovoltaic parameters of PSMs prepared with the blade-coating method. (**a**) Short-circuit current (I_{SC}), (**b**) V_{OC}, (**c**) FF, and (**d**) PCE.

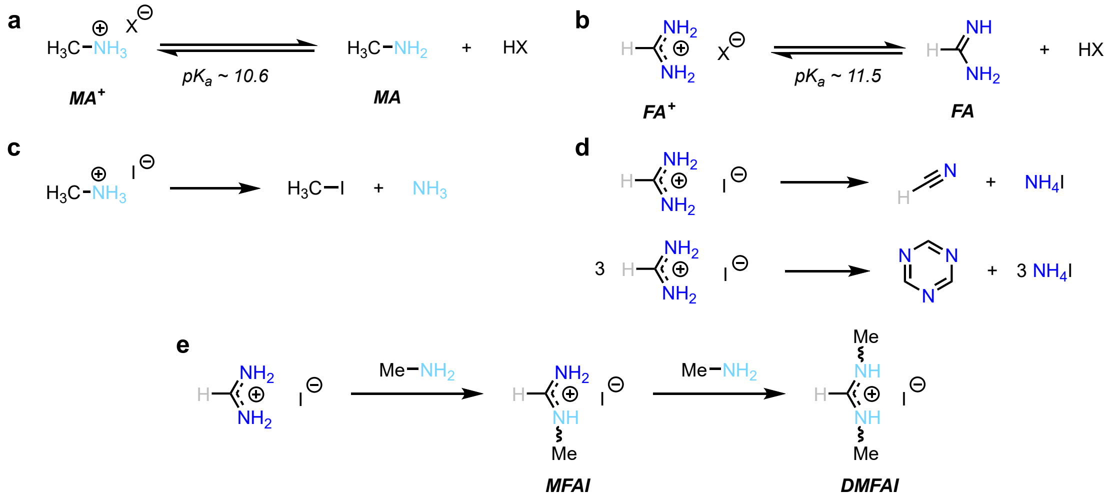

Supplementary Fig. S8 | Proposed pathways of relevant chemical decomposition of MA- and FA-based perovskites. Acid-base equilibrium of (**a**) MA^+ and (**b**) FA^+ cations. Decomposition reactions of (**c**) MAI and (**d**) FAI. (**e**) Condensation reaction between MA^+ and FA^+ cations in the hybrid perovskites.

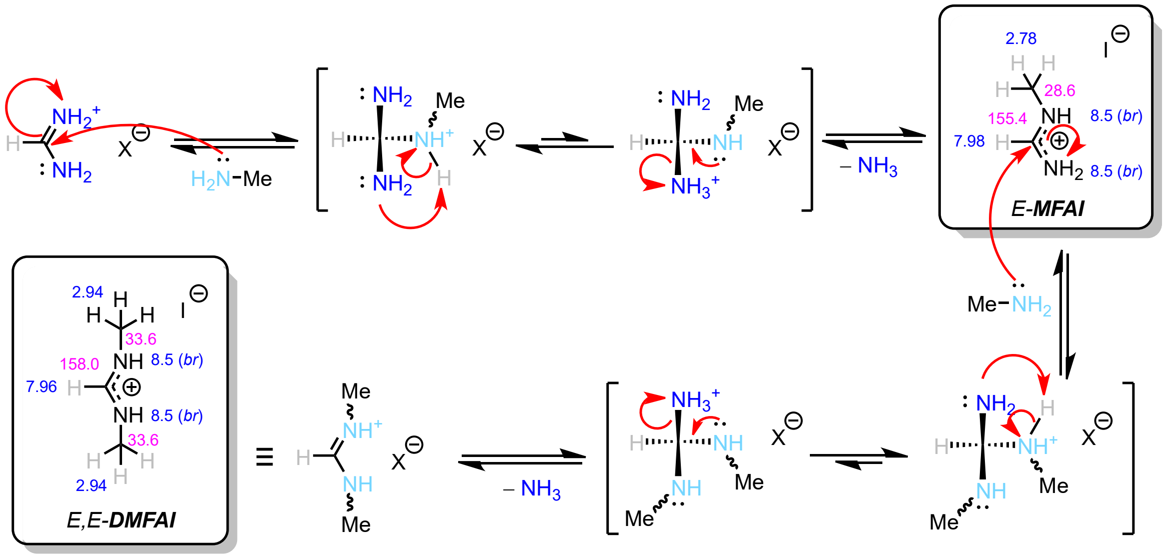

Supplementary Fig. S9 | Proposed mechanism of perovskite precursor solution (PPS) decomposition with assigned NMR chemical shifts of the formed species in the d_6-DMSO:d_7-DMF mixture (1:4, v/v). Chemical shifts for ^{1}H NMR in blue and for ^{13}C NMR in pink.

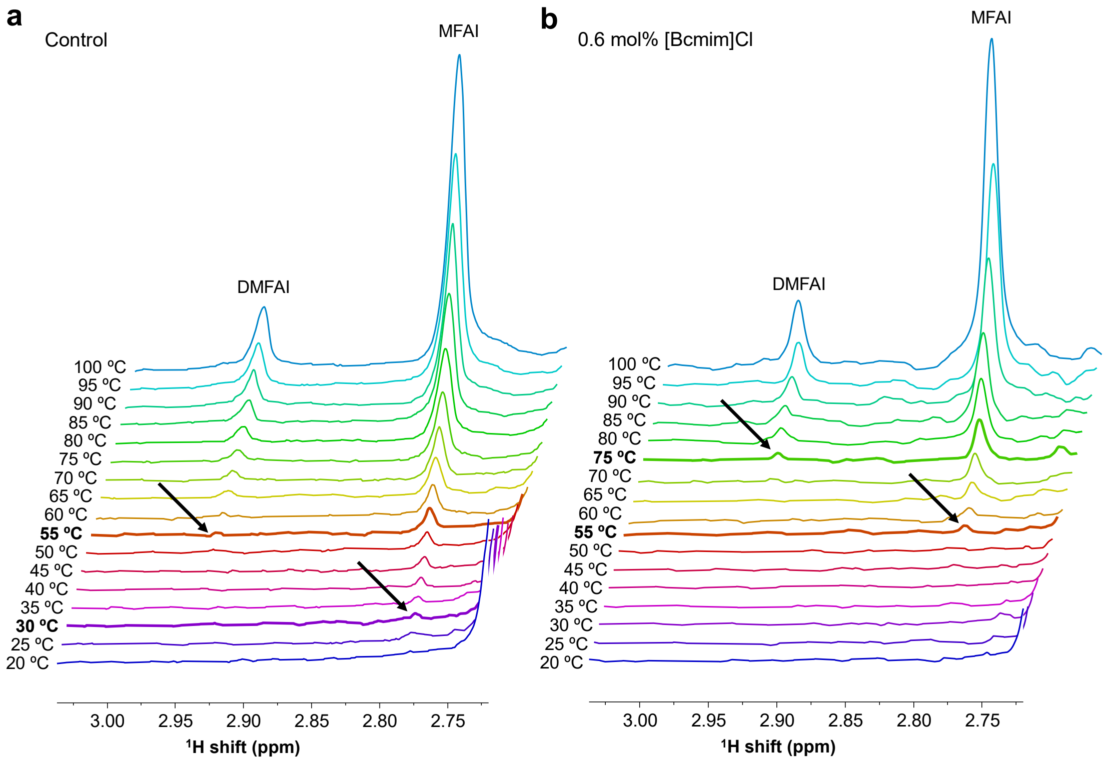

Supplementary Fig. S10 | ^{1}H NMR spectra of the (a) control and (b) target PPSs at different temperatures to study decomposition over time. Key peaks of DMFAI (2.94 ppm) and MFAI (2.78 ppm), and decomposition temperatures are highlighted.

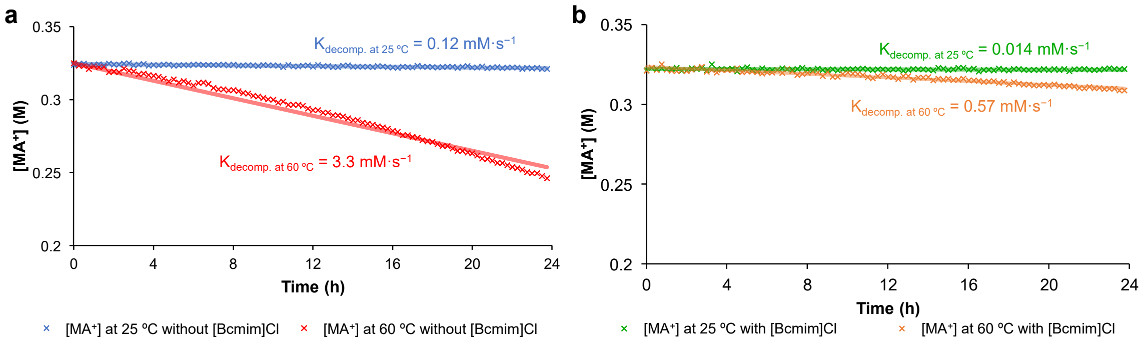

Supplementary Fig. S11 | Kinetic apparent rate constants for MA^+ decomposition as monitored by *in operando* NMR during the PPS stability study. (**a**) The control PPS at 25 ºC and 60 ºC; (**b**) The target PPS at 25 ºC and 60 ºC. Note: The calculated apparent 1st order rate constants of MA^+ decomposition was slower in the presence of [Bcmim]Cl (0.014 $mM{\cdot}s^{-1}$ at 25 ºC and 0.57 $mM{\cdot}s^{-1}$ at 60 ºC), compared to the control PPS (0.12 $mM{\cdot}s^{-1}$ and 3.3 $mM{\cdot}s^{-1}$ at 25 ºC and 60 ºC, respectively). In the control PPS, 27% of its initial MA^+ had decomposed after 24 h at 60 ºC *vs.* only 6% in the target PPS.

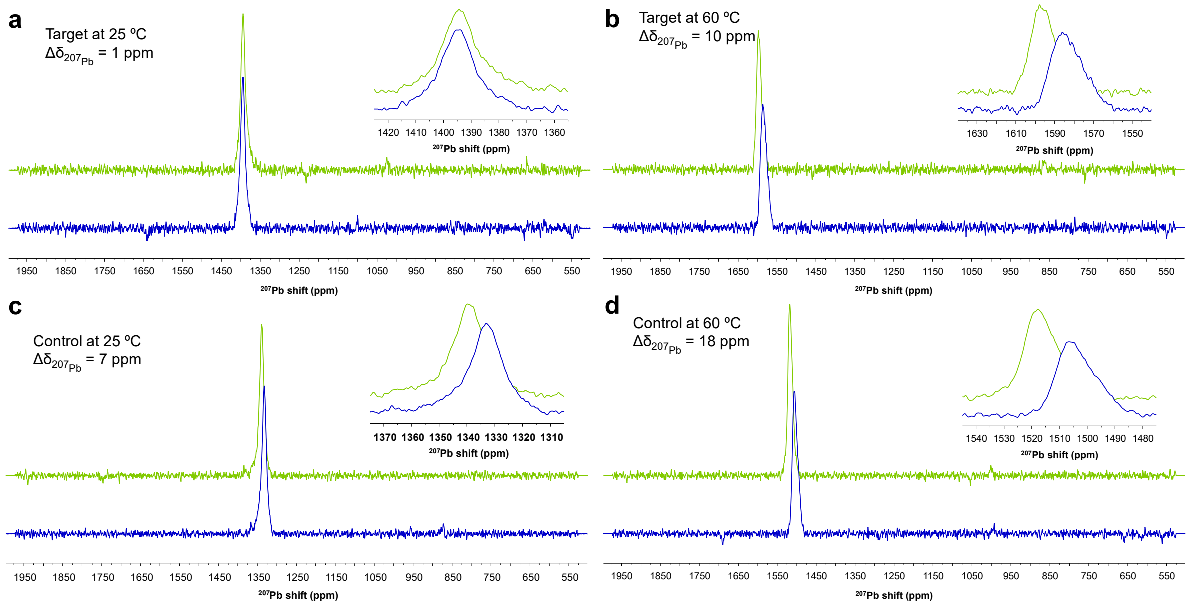

Supplementary Fig. S12 | ^{207}Pb NMR of the PPS at *t* = 0 h (blue) and *t* = 24 h (green) during the *in operando* NMR stability study. (**a**) The target PPS at 25 ºC; (**b**) The target PPS at 60 ºC; (**c**) The control PPS at 60 ºC; (**d**) The control PPS at 60 ºC. The inset plot is the relevant ^{207}Pb NMR peak. Note: ^{207}Pb solution NMR is highly sensitive to changes in chemical environment (*e.g.*, coordination or concentration) and temperature. Since other cations present in the PPS system are solvated and only interact with lead nuclei by changing the dielectric properties of the solution, studying ^{207}Pb peak shifts in situ can be used to probe changes in coordinating anion or halide concentration. In this system, downfield shifts between the *t* = 0 (blue) and *t* = 24 h (green) spectra in Supplementary Fig. S12 are indicative of higher free I^- concentration, attributed to the decomposition of the organic cations.

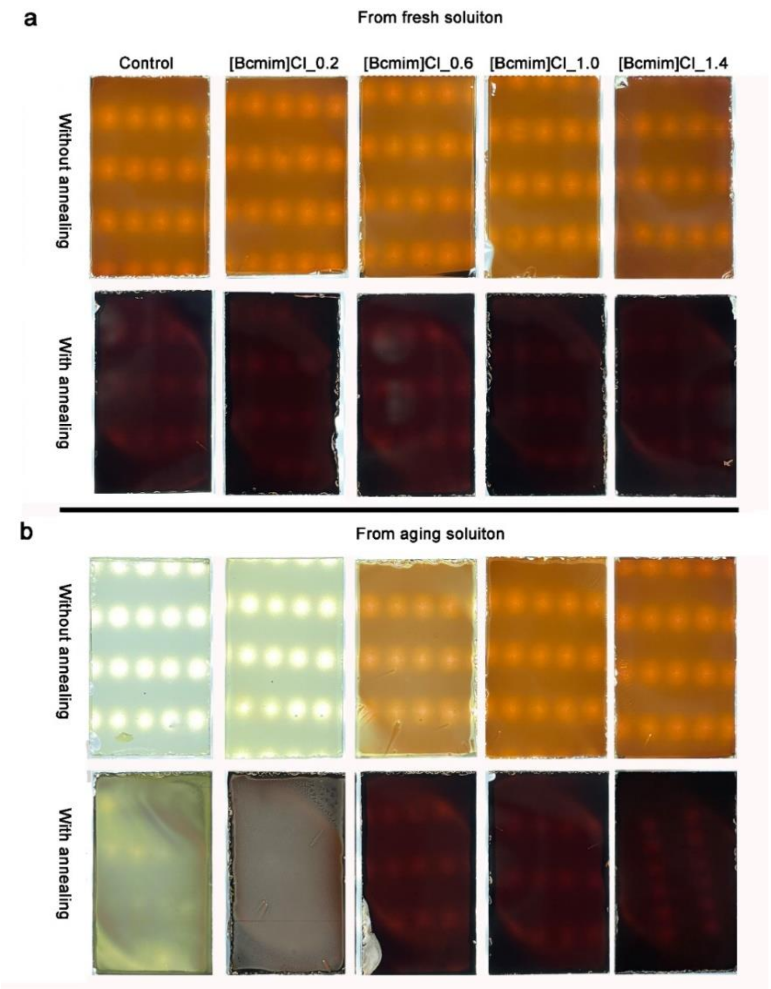

Supplementary Fig. S13 | Effect of perovskite precursor solution aging on perovskite films. (**a**) The unannealed and annealed films made with fresh perovskite precursor solutions with varying concentrations of [Bcmim]Cl. (**b**) The unannealed and annealed films made with 10-day aged perovskite precursor solutions with varying concentrations of [Bcmim]Cl. Note: All fresh perovskite precursor solutions containing [Bcmim]Cl and MACl were able to form black-phase $FAPbI_3$ perovskite, regardless of [Bcmim]Cl concentration (Supplementary Fig. S13a). The perovskite films made from aged perovskite precursor control solution (no [Bcmim]Cl) were light yellow in colour before annealing and contained undesirable yellow phase perovskite after annealing (Supplementary Fig. S13b). However, the inclusion of just 0.2 mol% [Bcmim]Cl in the perovskite precursor solution before aging for 10 days resulted in an annealed perovskite film with desirable black phase perovskite, albeit rough morphology. With more than 0.6 mol% [Bcmim]Cl concentration in the precursor solution, the perovskite films showed same morphology when prepared from fresh and aged precursor solutions.

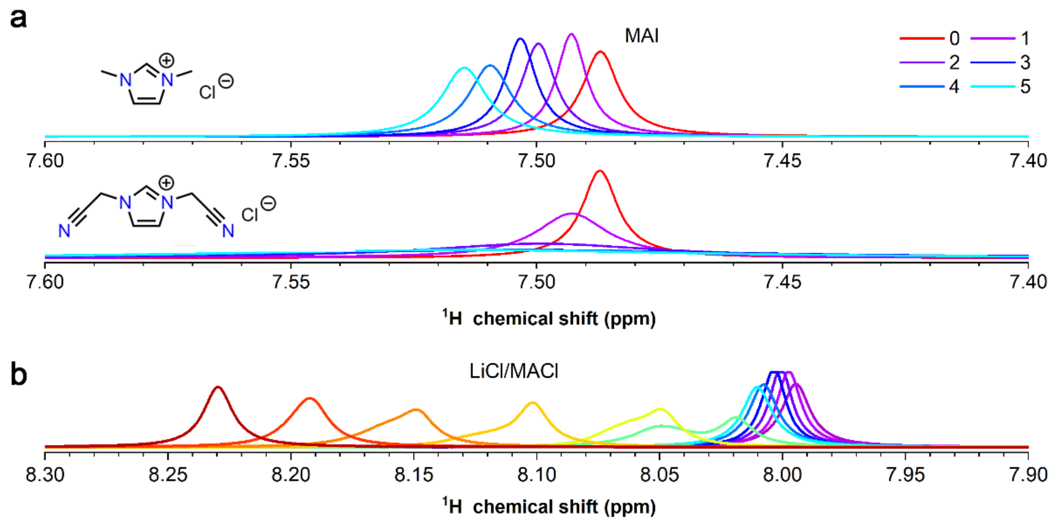

Supplementary Fig. S14 | NMR studies of the interaction between [Bcmim]Cl and MAX (X = Cl, I). (**a**) ^{1}H NMR of MAI solution as function of [Dmim]Cl or [Bcmim]Cl concentration. (**b**) ^{1}H NMR of MACl solution as function of LiCl concentration.

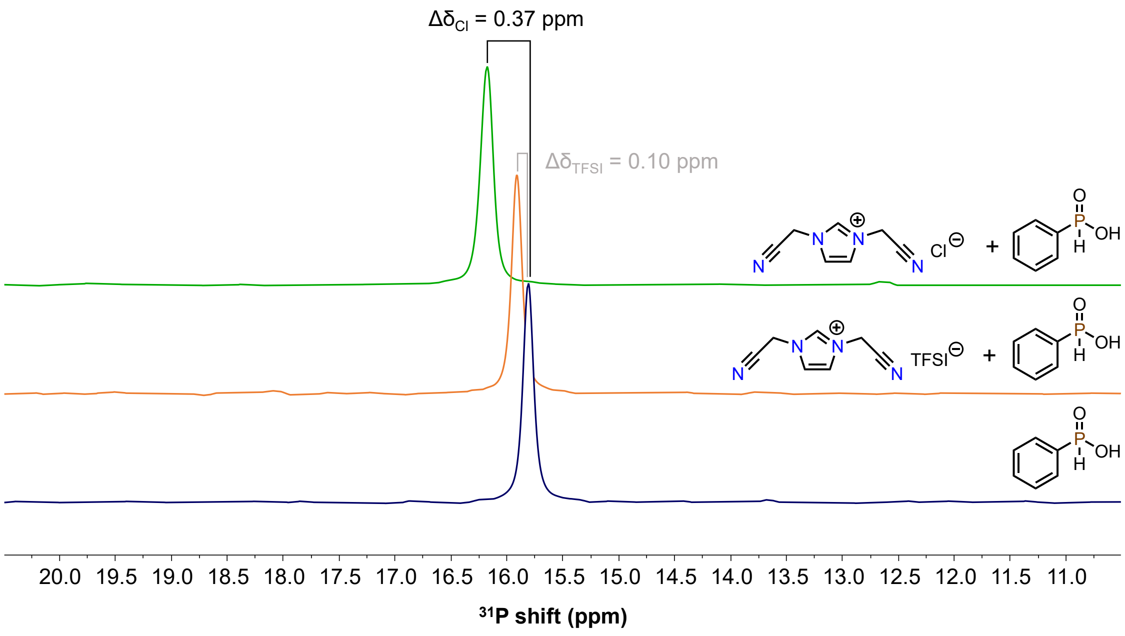

Supplementary Fig. S15 | Comparison of the HBA strength of [Bcmim]Cl and [Bcmim]TFSI. ^{31}P NMR spectra of [Bcmim]Cl with phenylphosphinic acid (top); [Bcmim]TFSI with phenylphosphinic acid (middle); Pure phenylphosphinic acid (bottom). Note: The ^{31}P NMR reported uses phenylphosphinic acid as a probe to quantify the HBA strength of different molecules. In this study, it is only used as a relative comparison tool and not an absolute comparison method.

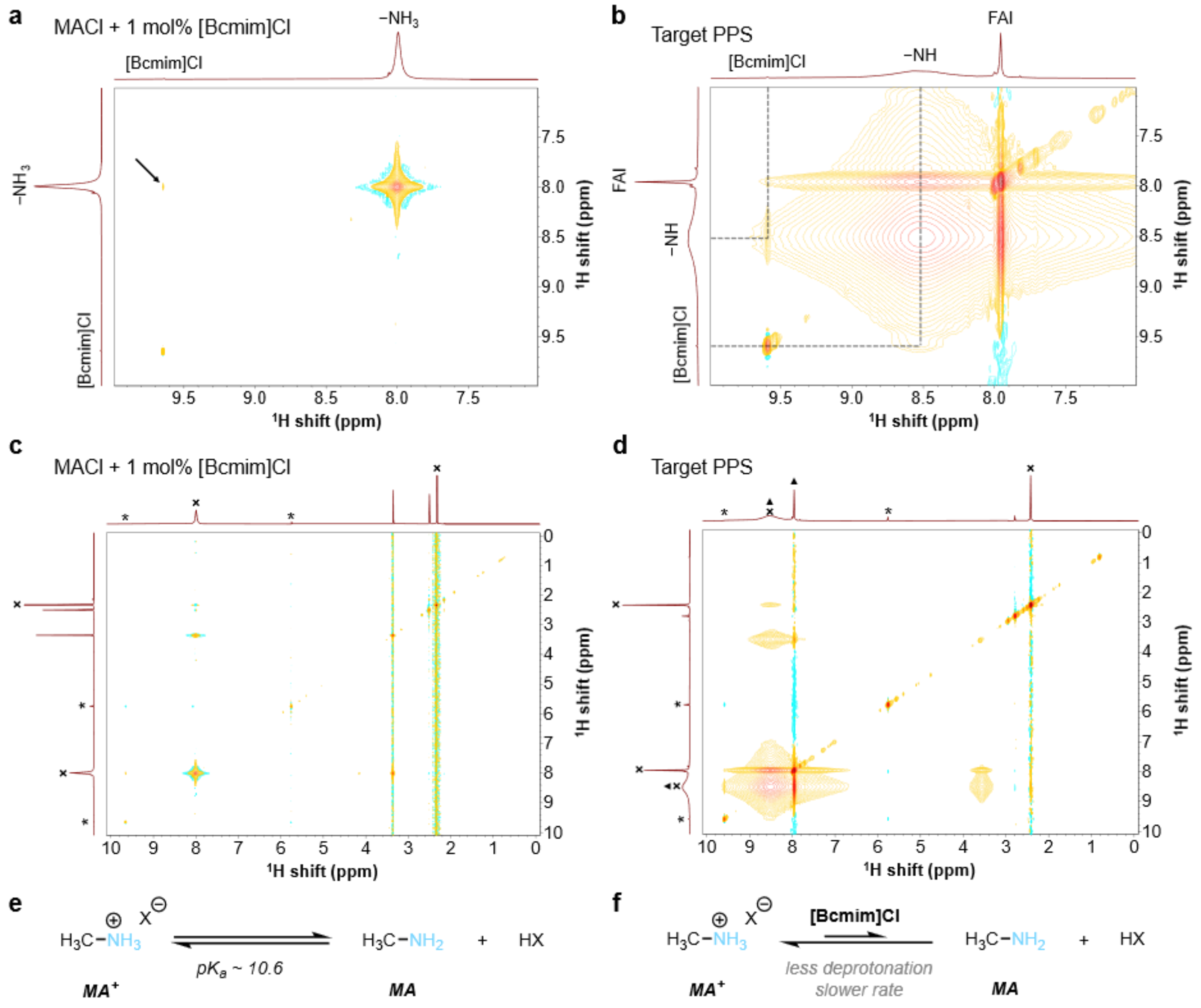

Supplementary Fig. S16 | Study of the [Bcmim]Cl acidic proton chemical exchange in the model solution and the target PPS. EXSY NMR spectra of (**a**) MACl + 1 mol% [Bcmim]Cl (400% zoom in the NH region shows a positive-phase cross peak coupling between the acidic proton of $[Bcmim]^+$ and the NH protons of MACl); (**b**) The target PPS (400% zoom in the NH region shows a positive-phase cross peak coupling between the acidic proton of $[Bcmim]^+$ and the NH protons in the PPS); (**c**) MACl + 1 mol% [Bcmim]Cl (full range); (**d**) The target PPS (full range). [Bcmim]Cl peaks are indicated with *, MA^+ by ×, and FA^+ by ▲. (**e**) Acid-base equilibrium of MA^+ and (**f**) possible chemical exchange interaction between [Bcmim]Cl and MA^+. Note: Exchange Spectroscopy (EXSY) is a 2D NMR experiment that can detect chemical exchange.

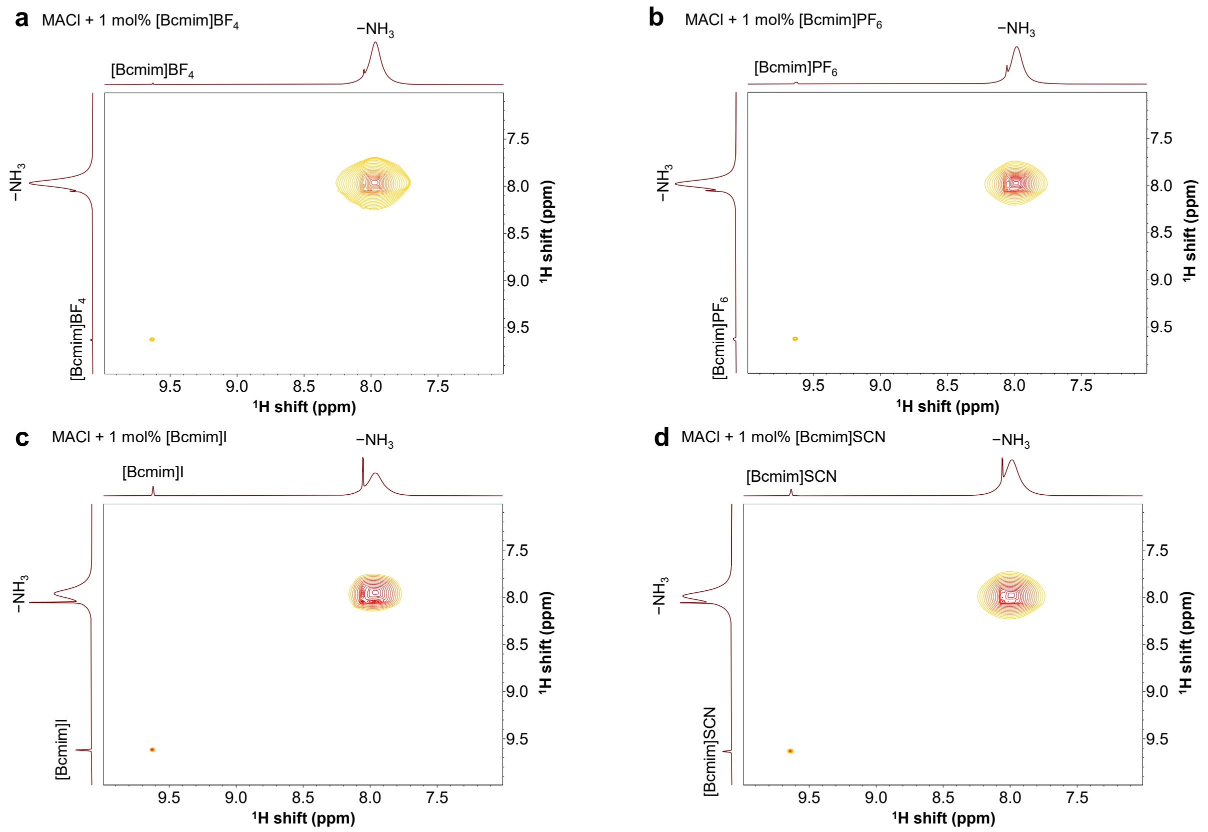

Supplementary Fig. S17 | Study of the [Bcmim]X acidic proton chemical exchange in the model solution. EXSY NMR spectra of MACl + (**a**) 1 mol% [Bcmim]BF_4, (**b**) 1 mol% [Bcmim]PF_6, (**c**) 1 mol% [Bcmim]I, and (**d**) 1 mol% [Bcmim]SCN. 400% zoom in the NH region.

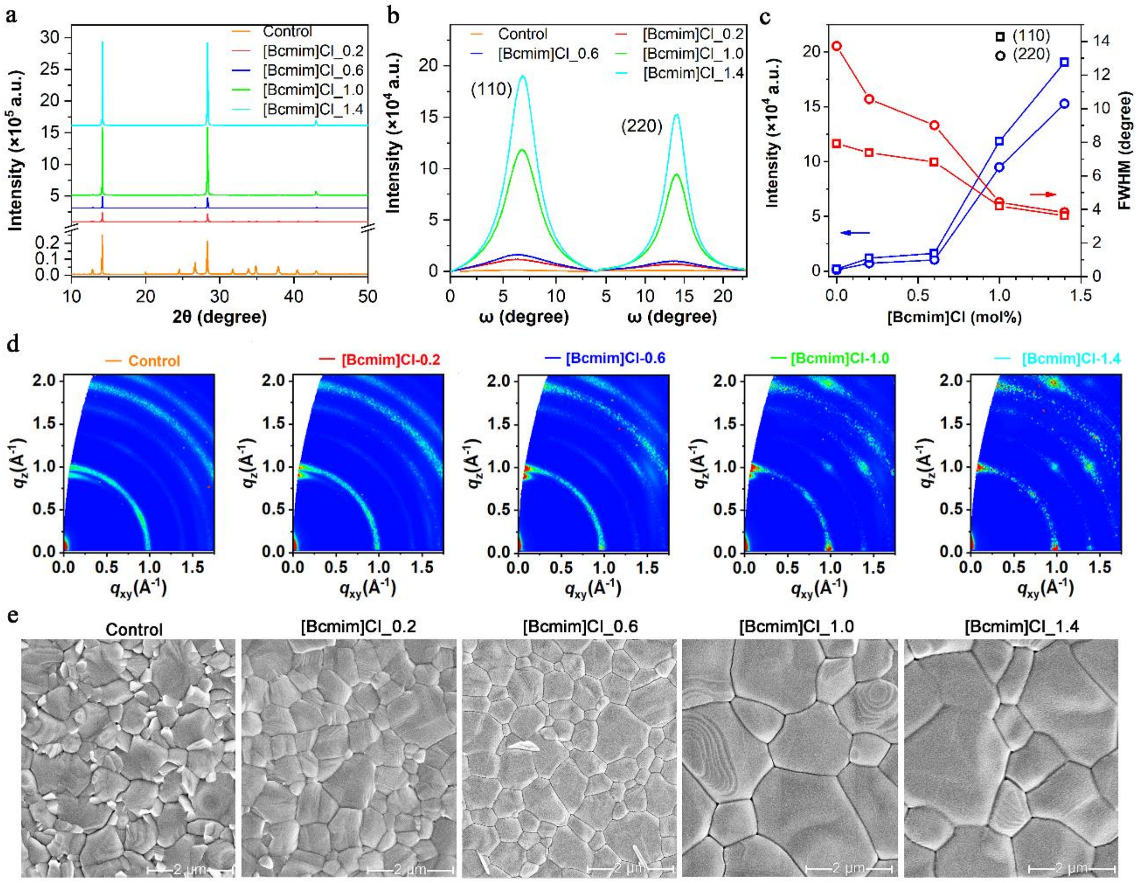

Supplementary Fig. S18 | Perovskite film characterisation. (**a**) XRD patterns of annealed perovskite films as a function of [Bcmim]Cl concentration. (**b**) Rocking curves produced by the (110) and (220) diffraction patterns of annealed perovskite films as a function of [Bcmim]Cl concentration. (**c**) The corresponding intensity and FWHM from the rocking curves of annealed perovskite films as a function of [Bcmim]Cl concentration. (**d**) GIWAXS spectra of annealed perovskite films as a function of [Bcmim]Cl concentration. (**e**) Top-view SEM images of annealed perovskite films as a function of [Bcmim]Cl concentration. Note: Different concentrations (0, 0.2, 0.6, 1.0, and 1.4 mol%) of [Bcmim]Cl were added into the perovskite precursor and labeled as "Control", "[Bcmim]Cl_0.2", "[Bcmim]Cl_0.6", "[Bcmim]Cl_1.0", and "[Bcmim]Cl_1.4", respectively. As shown in Supplementary Fig. S18a, all the perovskite films show similar diffraction peaks, but their intensities increase with increasing concentration of [Bcmim]Cl, indicating that the [Bcmim]Cl does not enter into the crystal lattice but instead promotes the crystallisation of the perovskite films. Notably, the preferential (110) orientation was observed in the rocking curve (Supplementary Fig. S18b), and the (110) and (220) facets

of the azimuthal scan profiles become higher and sharper with a reduced FWHM as the concentration of [Bcmim]Cl is increased (Supplementary Fig. S18c). Grazing incidence wide-angle X-ray (GIWAXS) scattering pattern of the 'Control' film shows a Debye-Scherrer ring of (110) facet ($q \approx 1.0$ $Å^{-1}$) with an almost isotropic intensity distribution along the azimuthal angle, exhibiting random arrangement of the crystal grains. In comparison, the discrete Bragg spots become clearer with increasing [Bcmim]Cl content.

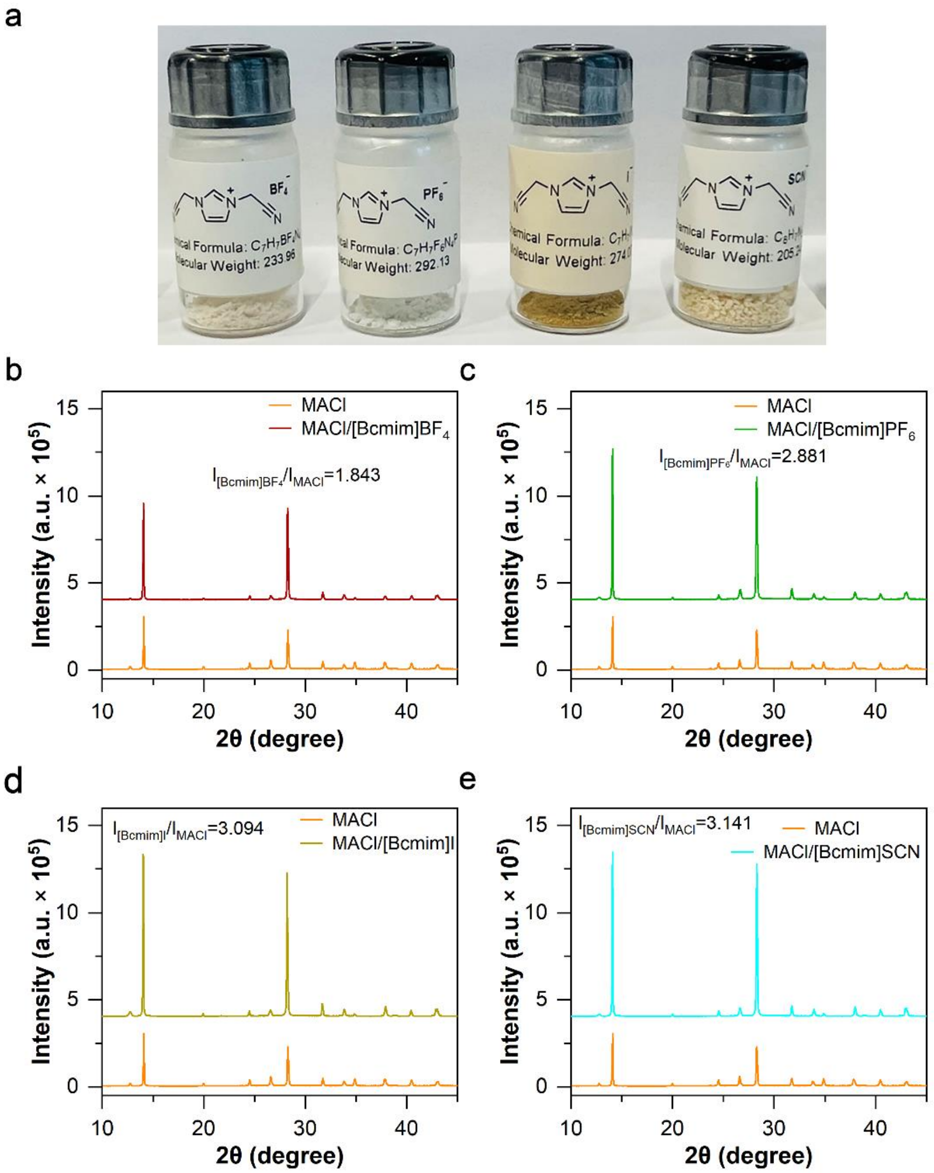

Supplementary Fig. S19 | XRD patterns of the perovskite films with or without ionic liquid after annealing at 100 °C for 60 min and at 150 °C for 10 min. (**a**) Photograph of the [Bcmim]X additives. (**b**) [Bcmim]PF_6, (**c**) [Bcmim]BF_4, (**d**) [Bcmim]I, and (e) [Bcmim]SCN compared to the control film. Note that regardless of anion, all [Bcmim]X (X = PF_6^-, BF_4^-, I^-, and SCN^-) additives enhanced the XRD intensity of the perovskite films (by 1.8, 2.9, 3.1 and 3.1 fold, respectively), significantly improving crystallinity compared to the control. This data demonstrates the beneficial role of the $[Bcmim]^+$ cation in enhancing device efficiency.

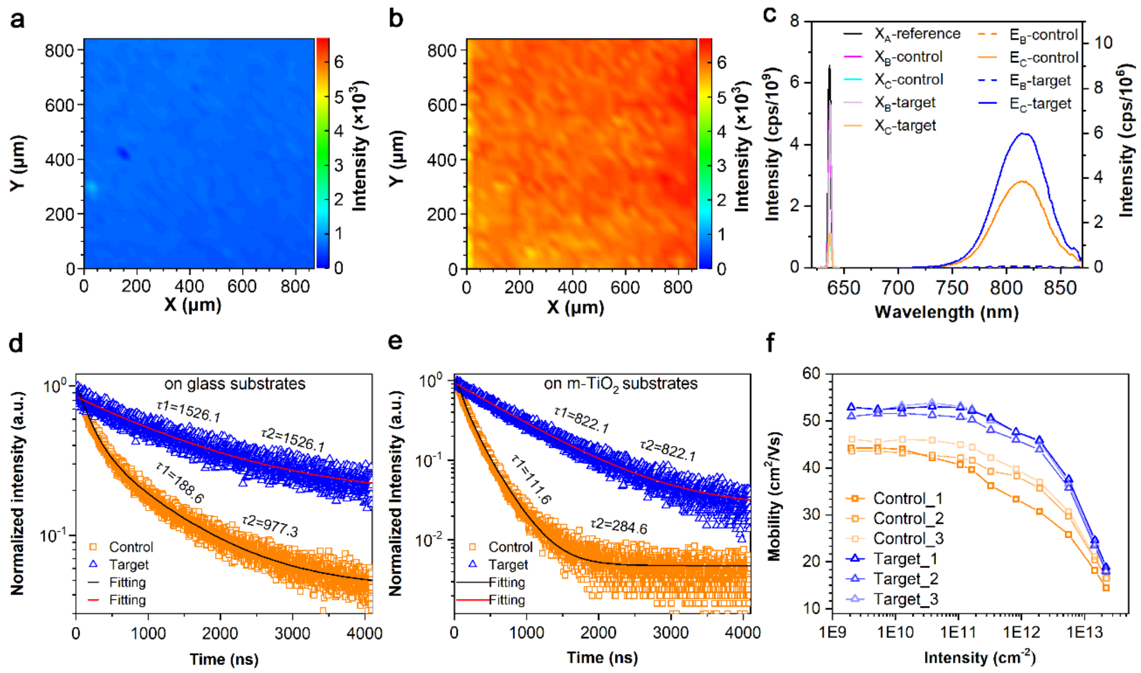

Supplementary Fig. S20 | Charge transfer dynamics of perovskite films. (**a**) Photoluminescence (PL) mapping image of the control film on a quartz glass. (**b**) Photoluminescence mapping image of the target film on a quartz glass. (**c**) Photoluminescence quantum yield of the control and target films on quartz glasses. (**d**) Time-resolved photoluminescence (TRPL) spectra of the control and target films on quartz glasses. (**e**) Time-resolved photoluminescence (TRPL) spectra of the control and target films on FTO/c-TiO_2/m-TiO_2 substrates. (**f**) Charge carrier mobility of the control and target films. Note: Steady-state PL mapping and TRPL measurements were performed to elucidate the effect of [Bcmim]Cl on the carrier lifetime of the perovskite films. Compared with the control film, the target film shows a uniform map and has much higher intensity, suggesting lowered non-radiative recombination (Supplementary Figs. S20a and S20b). The photoluminescence quantum yield (PLQY) of control and target films is 1.36% and 2.33%, respectively (Supplementary Fig. S20c). The enhanced PLQY of the target film is attributed to the improved crystallinity and reduced trap densities. As shown in Supplementary Figs. S20d and S20e, the target film gives a longer lifetime of 1526.1 ns on a quartz substrate (822.1 ns on FTO/*c*-TiO_2/*m*-TiO_2 substrates), which is much higher than that of 977.3 ns (284.6 ns on FTO/*c*-TiO_2/*m*-TiO_2 substrates) of the control film. It was also found that the average carrier mobility of the Target films is 52.3 cm^2/Vs, which is higher than that of the control film with a value of 44.6 cm^2/Vs

(Supplementary Fig. S20f). Combined with carrier mobility and lifetime, the calculated carrier diffusion length (L_D) of control films on the quartz glass and mesoporous substrates is 10.6 and 5.7 μm, which are shorter than that of Target films (14.4 and 10.5 μm) (Supplementary Table S2). The prolonged lifetime and carrier diffusion length of the target film indicate suppressed trap-assisted non-radiative recombination within the devices, which is in agreement with the enhanced V_{OC}.

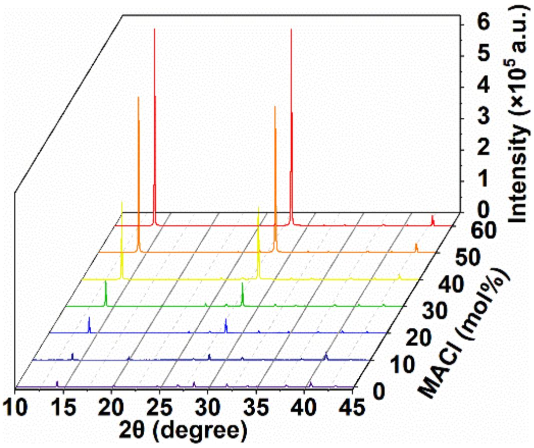

Supplementary Fig. S21 | XRD patterns of $Cs_{0.05}MA_{0.05}FA_{0.90}PbI_3$ perovskite films with varying amounts of MACl in the perovskite precursor annealed at 100 ºC for 1 h.

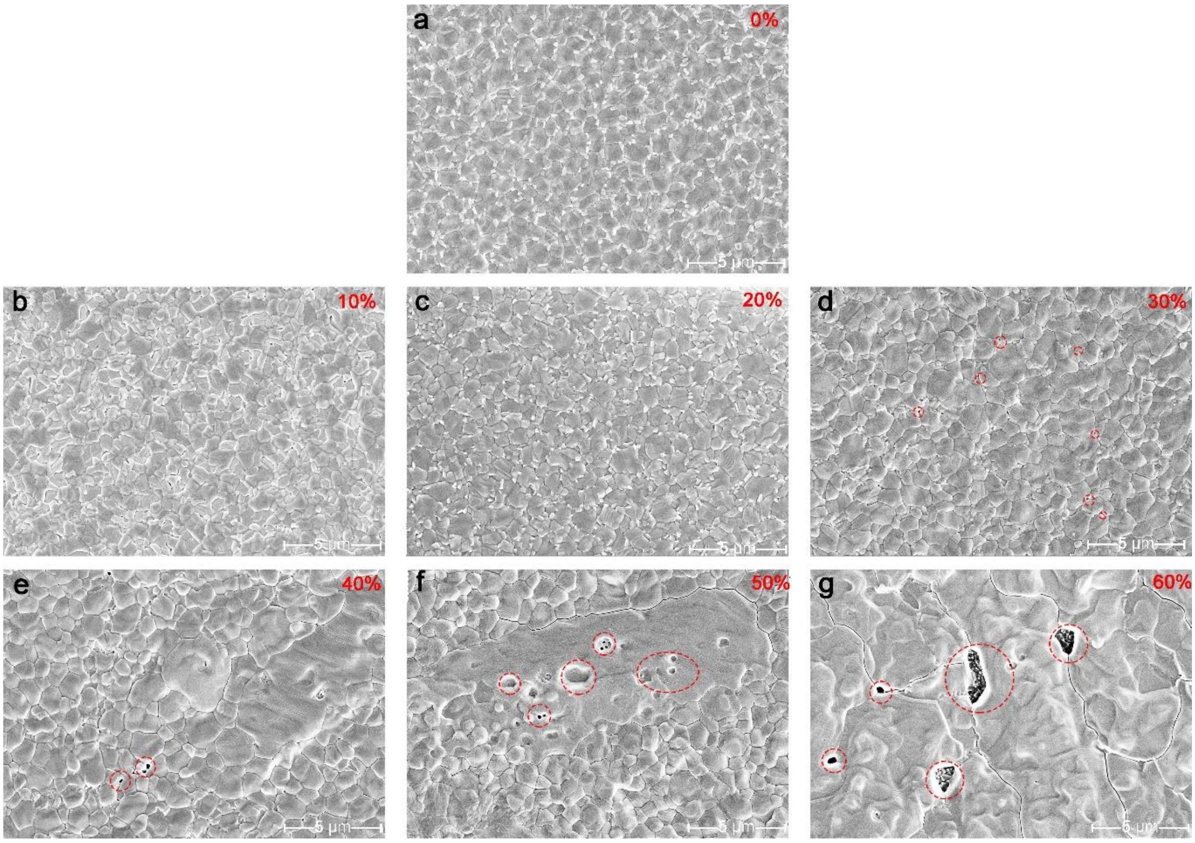

Supplementary Fig. S22 | Top-view SEM images of $Cs_{0.05}MA_{0.05}FA_{0.90}PbI_3$ perovskite films with varying MACl concentrations. (**a**) The pristine film, (**b**) 10 mol%, (**c**) 20 mol%, (**d**) 30 mol%, (**e**) 40 mol%, (**f**) 50 mol%, and (**g**) 60 mol% of MACl doped into the perovskite films. Note: Red circles label pinholes and voids in films. The SEM images show a clear trend of increased grain sizes with increasing MACl content. For MACl concentrations ≤ 20 mol%, the perovskite films exhibit uniform morphology with a full coverage (Supplementary Figs. S22a-S22c). When the concentration of MACl is above 30 mol%, pinholes between grains in the perovskite films appear (Supplementary Fig. S22d). At even higher concentrations of MACl, the perovskite films exhibit poor coverage with a lot of pinholes or voids (Supplementary Figs. S22e-S22g). The formation of void and/or pinholes is presumably related to the rapid release of MACl. Such voids mostly cause shunts, resulting in low performance and poor stability.

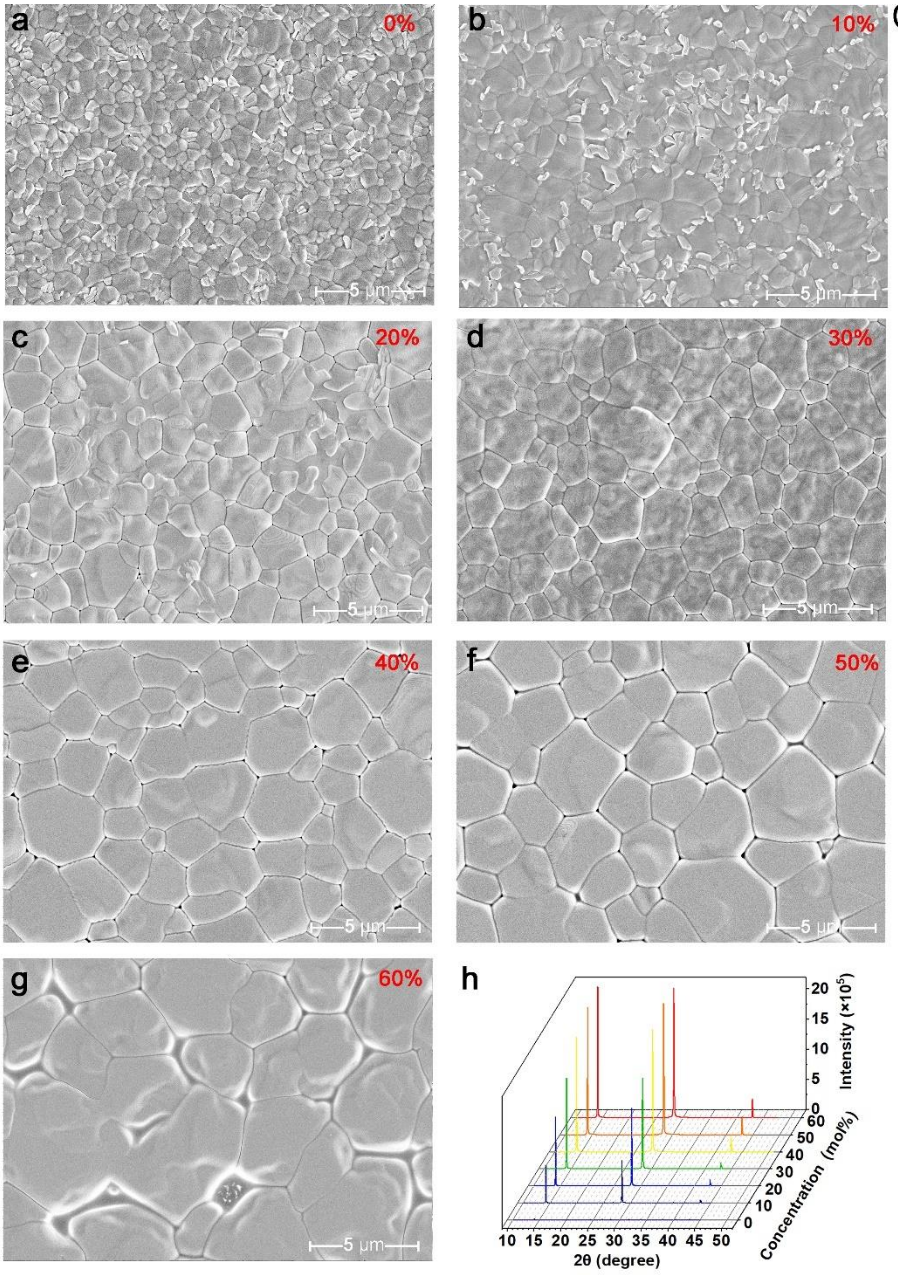

Supplementary Fig. S23 | Top-view SEM images of perovskite films (containing 0.6 mol% [Bcmim]Cl) with varying MACl concentrations. (**a**) The pristine film, (**b**) 10 mol%, (**c**) 20 mol%, (**d**) 30 mol%, (**e**) 40 mol%, (f) 50 mol% and (g) 60 mol% of MACl doped into the perovskite films. (**h**) The corresponding XRD patterns perovskite films annealed at 100 ºC for 1 h.

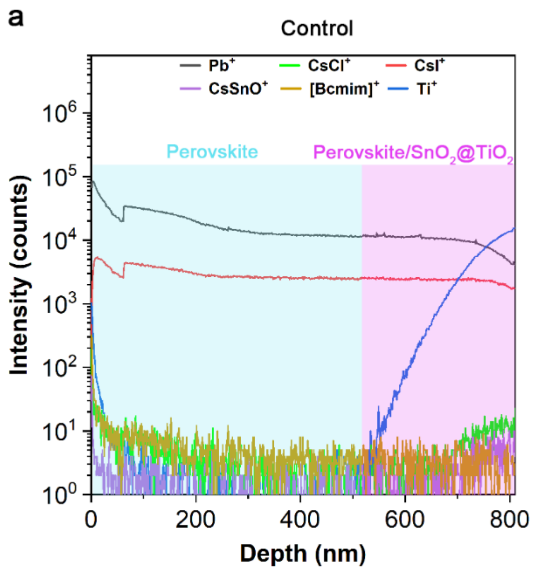

Supplementary Fig. S24 | ToF-SIMS depth profiling of the perovskite films. (**a**) Control and (**b**) target perovskite/SnO_2@TiO_2/FTO films. The [Bcmim]$^+$ signal is located in the bulk and surface of the perovskite film, but mostly accumulates at the interface between the SnO_2@TiO_2 layer and the perovskite layer.

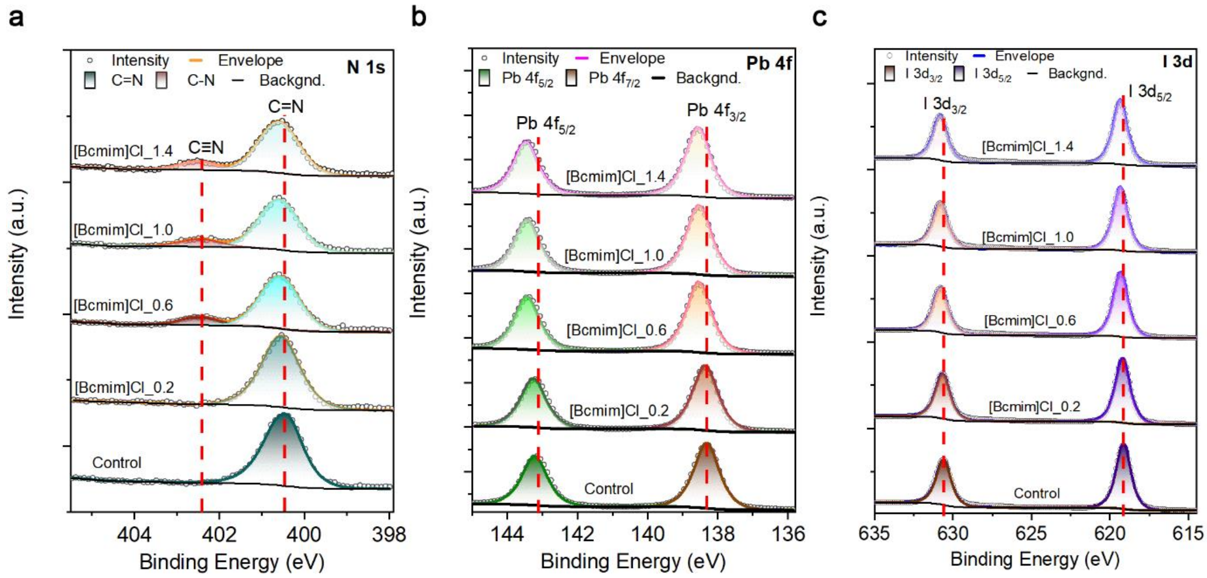

Supplementary Fig. S25 | High-resolution XPS spectra. (**a**) N 1s, (**b**) Pb 4f and (**c**) I 3d regions of the control and [Bcmim]Cl films. Note: The Pb core level spectra shift toward higher binding energy for the [Bcmim]Cl-modified perovskite film compared to the control sample, consequence of the changes in the electronic environment surrounding Pb due to substitution of I ($\chi = 2.66$) by the more electron-rich N ($\chi = 3.04$) and Cl ($\chi = 3.15$), indicating the existence of strong interaction between the [Bcmim]Cl and perovskite[1]. $[Bcmim]^+$ and Cl^- might interact with the negatively charged Pb-I antisites and the under-coordinated Pb^{2+} sites. The addition of [Bcmim]Cl is beneficial to passivate under-coordinated Pb^{2+} defects and lowers trap densities in the perovskite films, resulting in the enhanced device performance[2].

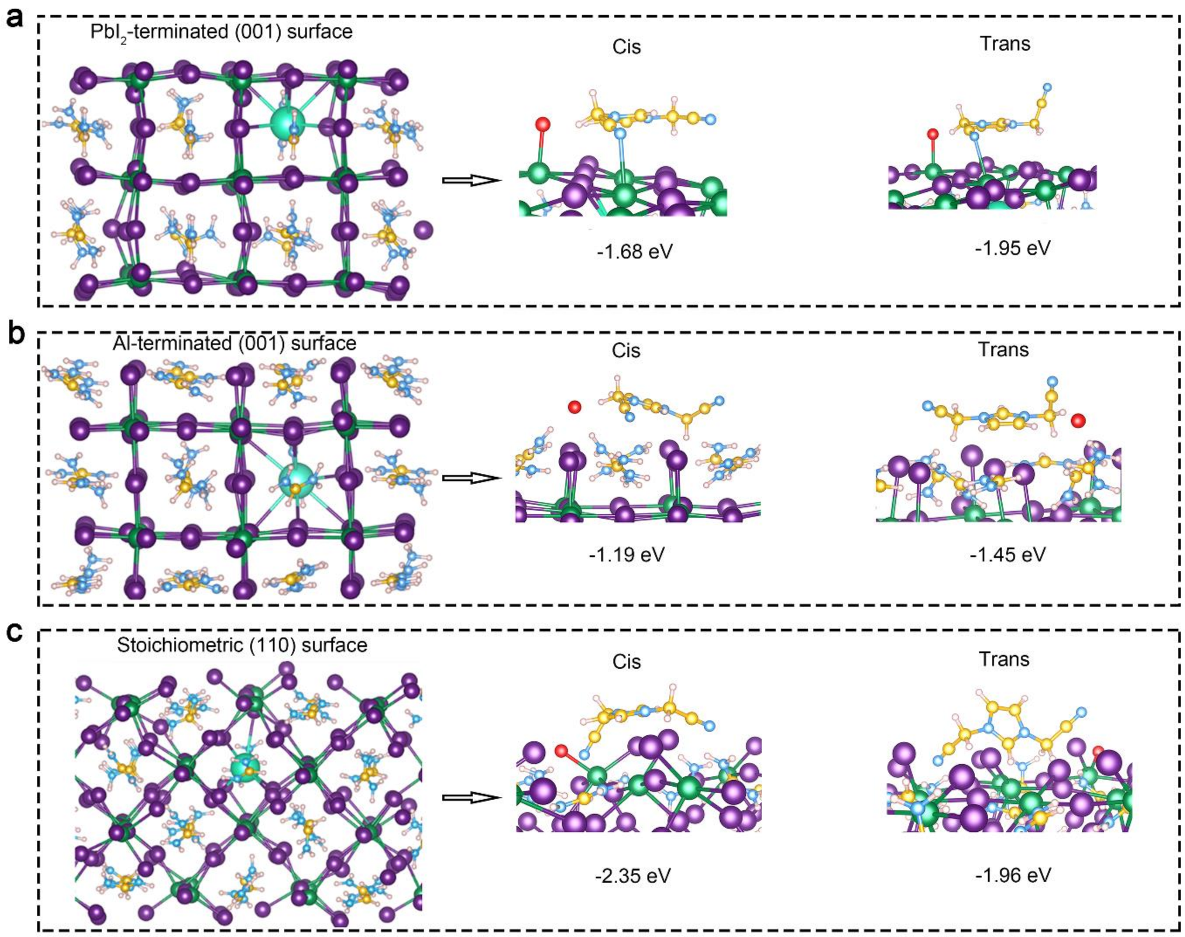

Supplementary Fig. S26 | Graphical representation of the results obtained from DFT calculations. (**a**) Structure of a generic undercoordinated lead crystal and of [Bcmim]Cl adsorbed on a PbI_2-terminated perovskite (001) surface. (**b**) Structure of a generic Al-terminated perovskite crystal and of [Bcmim]Cl adsorbed on the Al-terminated perovskite (001) surface. (**c**) Structure of a generic stoichiometric lead perovskite crystal and of [Bcmim]Cl adsorbed on a stoichiometric perovskite (110) surface. Note: Pb, I, Cs, C, N, H and Cl atoms are denoted in green, purple, cyan, yellow, blue, white and red spheres, respectively. Cis and Trans refer to the two possible $[Bcmim]^+$ isomers employed as initial conformations in the calculation, corresponding to the 0 and 180° N – C – C – N dihedral angles formed by nitrile-groups, respectively.

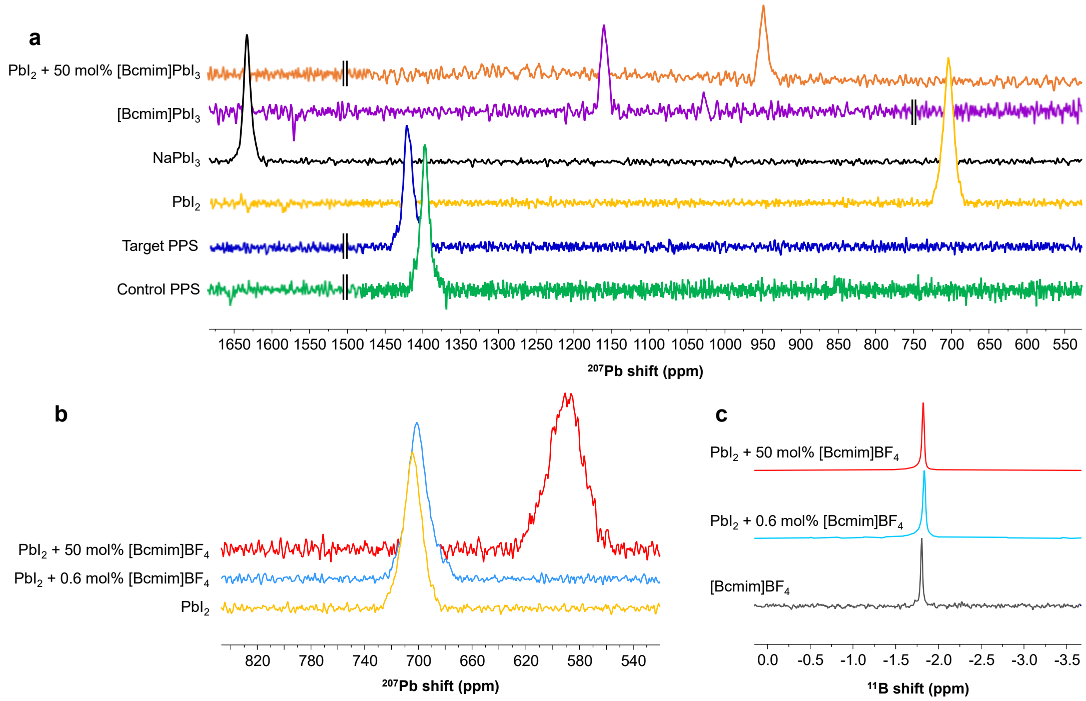

Supplementary Fig. S27 | ^{207}Pb solution NMR study of the interaction between nitrile groups in [Bcmim]$^{+}$ cations and lead. (**a**) ^{207}Pb NMR spectra of the control (green) and target (dark blue) PPS, PbI_2 (yellow), $NaPbI_3$ (black), [Bcmim]PbI_3 (purple), and an equimolar mixture of PbI_2 and [Bcmim]PbI_3 (orange); (**b**) ^{207}Pb NMR of PbI_2 (yellow) and 0.6 mol% [Bcmim]BF_4 (target ratio, light blue), and 50 mol% [Bcmim]BF_4 (equimolar, red). (**c**) ^{11}B NMR spectra of [Bcmim]BF_4 (gray), PbI_2 + 0.6 mol% [Bcmim]BF_4 (light blue), and PbI_2 + 50 mol% [Bcmim]BF_4 (red), where the lack of BF_4^- peak shift corroborates the absence of interaction between the lead and the tetrafluoroborate counterion. Note: ^{207}Pb solution NMR is highly sensitive to changes in chemical environment (*e.g.* coordination or concentration) and temperature. Therefore, assigning changes in the chemical shift to specific effects requires thorough control and referencing. As [Bcmim]$^{+}$ contains a coordinating halide as a counterion, peak shifts from [Bcmim]$^{+}$ influence can't be decoupled from the presence of additional halides. PbI_2 was used as reference. As shown in Supplementary Fig. S27a, $NaPbI_3$ was used as a control, selected to emulate solely the interaction between additional halide and lead, *viz.* PbI_3^-. As shown in Supplementary Fig. S27b, [Bcmim]BF_4 was used as the BF_4^- counter anion is non-coordinating and was selected to study solely the nitrile donor ability, *viz.* [Bcmim]$^{+}$, by decoupling any interaction between additional halide (from the ionic liquid) and lead.

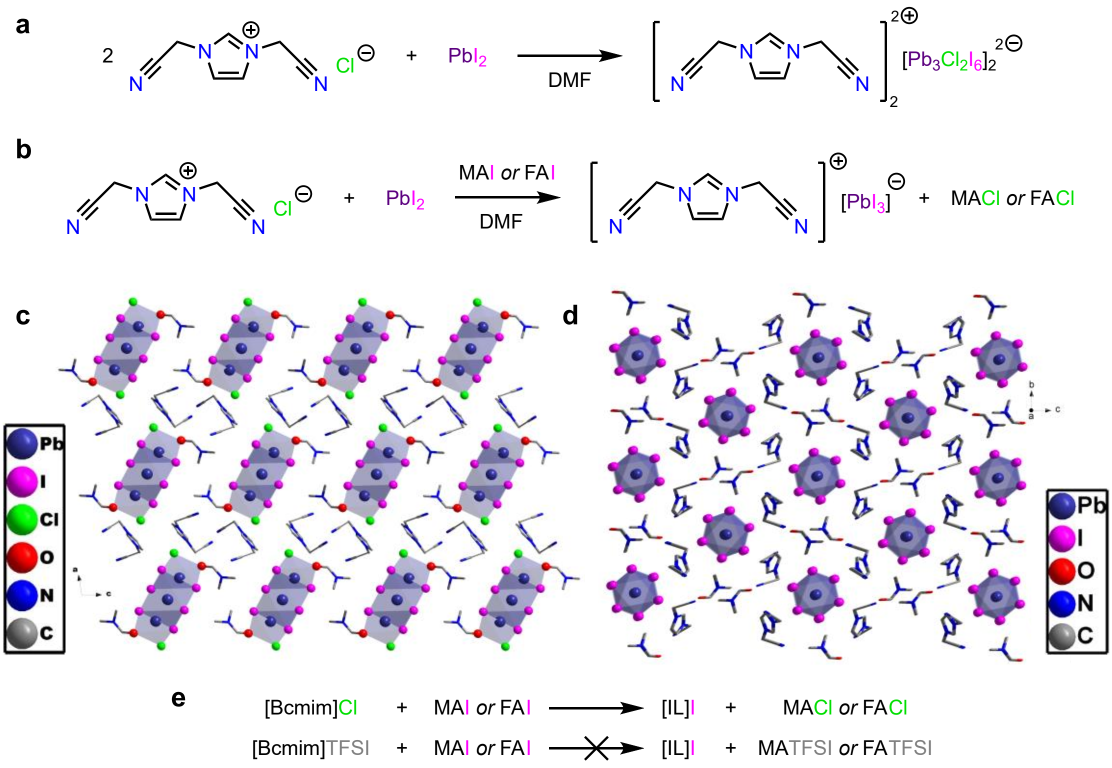

Supplementary Fig. S28 | Interaction of [Bcmim]Cl with perovskite precursor. (**a**) Scheme of the reaction between [Bcmim]Cl and PbI_2 (molar ratio=1:1) in DMF. (**b**) Scheme of the reaction between [Bcmim]Cl, PbI_2, and MAI (or FAI) (molar ratio=1:1:1) in DMF. (**c**) Packing illustration of [Bcmim]$_4$Pb$_3$Cl$_2$I$_6$·2DMF. (**d**) Packing illustration of [Bcmim]PbI$_3$·DMF. (**e**) Counterion exchange reactions between [Bcmim]X (Cl^-, $TFSI^-$) and MAI or FAI. Note: The reaction of [Bcmim]Cl with PbI_2 in a 1:1 molar ratio generates one-dimensional (1D) [Bcmim]$_4$Pb$_3$Cl$_2$I$_6$ (Supplementary Fig. S28a). When MAI (or FAI) is present, this reaction gives rise to [Bcmim]PbI$_3$, along with MACl (or FACl) (Supplementary Fig. S28b). Crystals were formed during the evaporation process, and the structure of the crystals was analyzed by SC-XRD (Supplementary Table S3), which show the 1D-polymeric nature of the anion (Supplementary Figs. S28c and S28d), and the inclusion of the DMF solvent into the crystal structure which could be easily lost at room temperatu

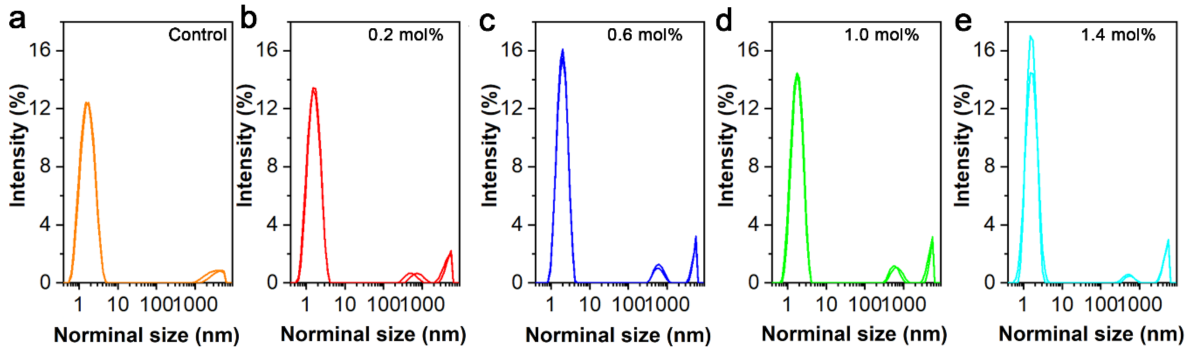

Supplementary Fig. S29 | DLS profile of the PPS with varying concentrations of [Bcmim]Cl. (**a**) The control, (**b**) 0.2 mol%, (**c**) 0.6 mol%, (**d**) 1.0 mol% and (**e**) 1.4 mol% of [Bcmim]Cl solution.

Note: DLS shows the statistical size distributions of the species in the PSS containing different concentrations (0-1.4 mol%) of [Bcmim]Cl (Supplementary Fig. S29). In the control PPS, the average diameter of the aggregates is 1.65 nm and 4380 nm with intensities of 12 and 1%, respectively. However, the average diameter distribution of aggregates for the target PPS has three peaks at 1.65 nm, 600 nm and 5000 nm with intensities of 14, 1 and 2%, respectively. The inclusion of [Bcmim]Cl in the perovskite precursor may induce the growth of more particles and promotes the formation of new smaller-sized aggregates. The model reactions between [Bcmim]Cl and other PPS components provide further evidence for the existence of additional aggregates in the form of one-dimensional salts (Supplementary Fig. S28).

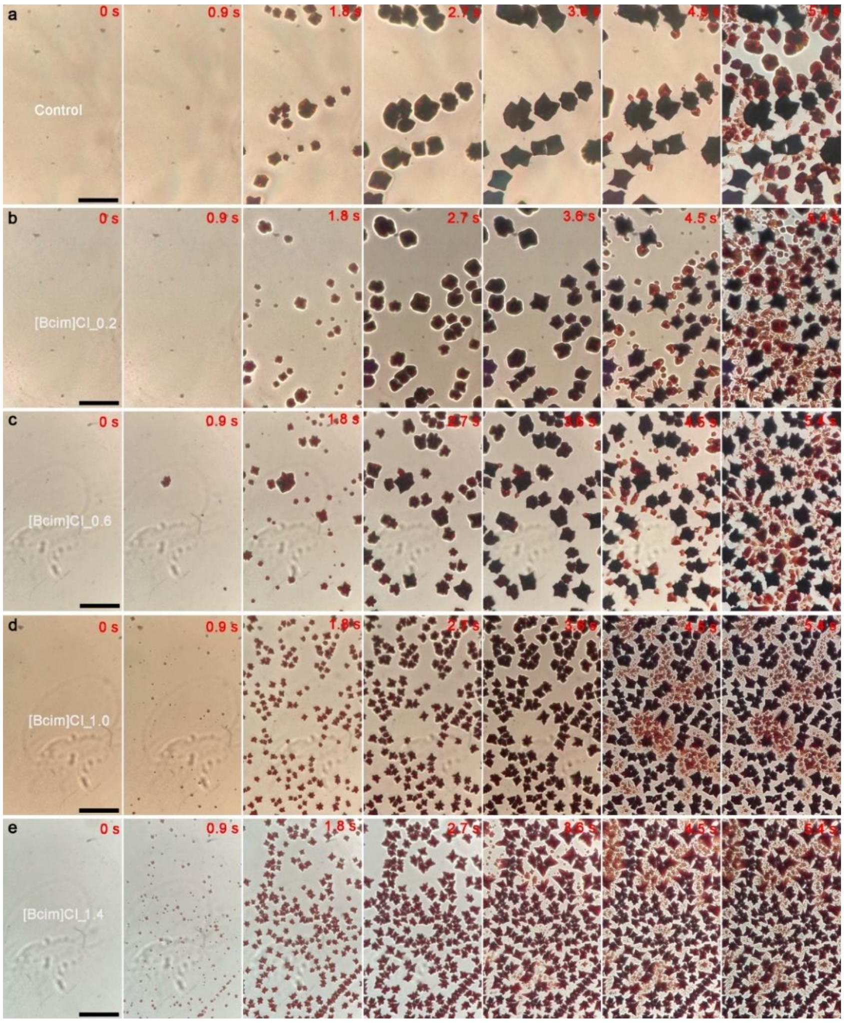

Supplementary Fig. S30 | Frames of the perovskite film crystal growth process from precursor (wet) perovskite films with varying concentrations of [Bcmim]Cl annealed at 100 ºC. (**a**) The control PPS, (**b**) 0.2 mol%, (**c**) 0.6 mol%, (**d**) 1.0 mol% and (**e**) 1.4 mol% of [Bcmim]Cl. The scale bar is 5 μm. Note: The individual frames were captured from a video recording of the drying process of wet perovskite films annealed at 100 ºC from 0 to 5.4 s. All films show similar growth behaviour in which a few seed crystals initially appear, which then grow and spread, while new crystal nuclei are constantly formed. With increasing [Bcmim]Cl concentration, more seed crystals appear in the same interval, indicating that [Bcmim]Cl concentration is positively correlated with nucleation rate. After 3.6 s, the grain size decreases as a function of the [Bcmim]Cl content. Therefore, the inclusion of [Bcmim]Cl in the precursor solution promotes perovskite crystal nucleation and slows down grain growth, which is in accordance with previous work[3].

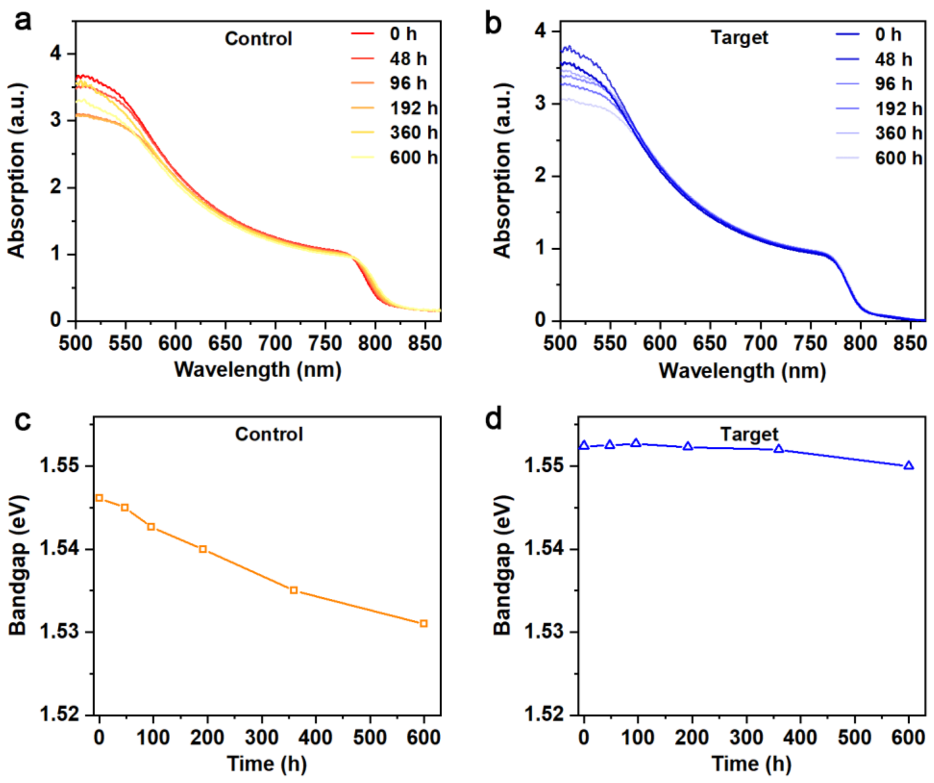

Supplementary Fig. S31 | Evolution of the perovskite film absorption spectra and bandgaps after ageing at 60 ± 5 ℃ for up to 600 h in a N_2 glove box. UV-vis absorption spectra of the (**a**) control and (**b**) target films as a function of time. The corresponding bandgap change of the (**c**) control and (**d**) target films as a function of time. Note: Both films show a decrease of absorption in the short wavelength region (< 550 nm) within the study timeframe. However, the control film shows a red shift of the absorption edge and severe band gap shrinkage during thermal aging, whereas the target film exhibits virtually little change of absorption edge and band gap.

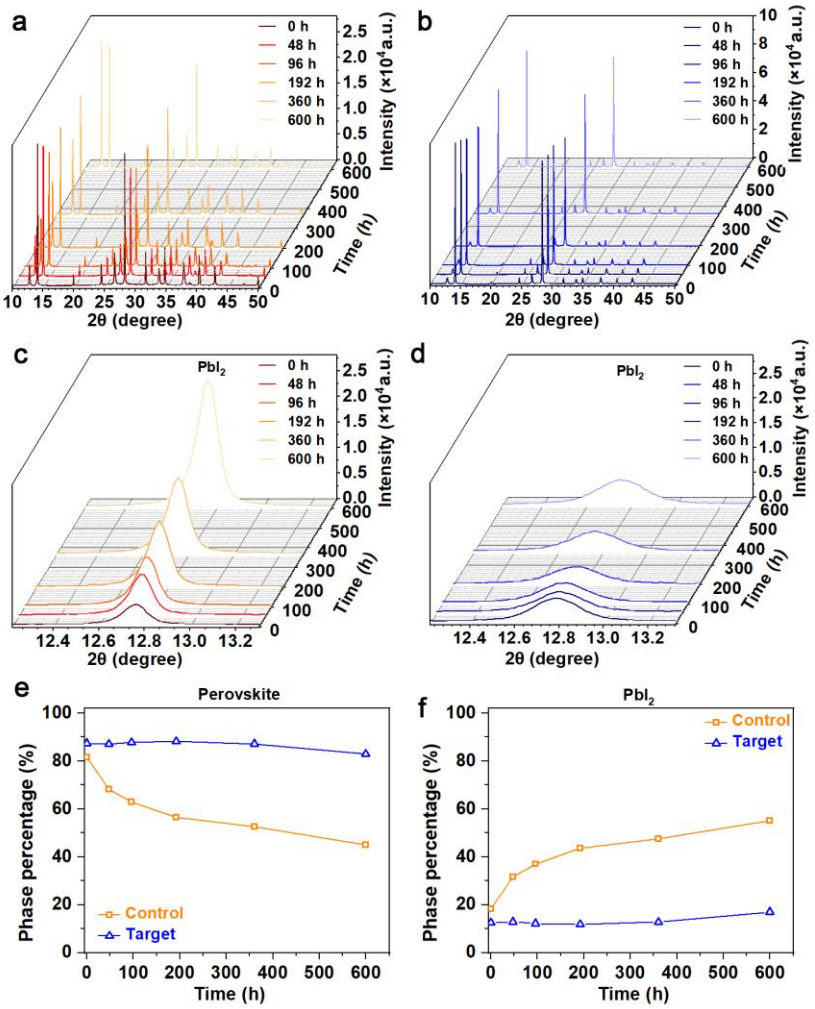

Supplementary Fig. S32 | Evolution of the phase composition of the control and target films after aging at 60 °C for up to 600 h in a N_2 glove box. (**a**) XRD patterns of the control film as a function of aging time. (**b**) XRD patterns of the target film as a function of aging time. (**c**) (001) peak of PbI_2 in the control film. (**d**) (001) peak of PbI_2 in the target film. (**e**) Change of the PbI_2 phase percentage as a function of aging time. (**f**) Change of the PbI_2 phase and perovskite 3C phase percentage as a function of aging time. Note: the XRD patterns reveal the formation of the PbI_2 phase (12.70°) before ageing for both the control (Supplementary Fig. S32a) and target (Supplementary Fig. S32b) films. However, the control film has a higher intensity of the PbI_2 phase over 600 h (Supplementary Fig. S32c) whereas the target film exhibits a minor change of intensity for the PbI_2 peak over 600 h (Supplementary Fig. S32d). The peak area of the PbI_2 phase ((001) peak) and perovskite 3C phase ((110) peak) in the films provides information about the decomposition rate of the perovskite films[4]. The phase percentages in the perovskite film were determined by calculating the integrated peak intensity

ratios of each selected phase peak area over the sum of all phases [5]. As shown in Supplementary Figs. S32e and S32f, PbI_2 in the control film increases, whereas the target film only shows a slight increase of PbI_2, demonstrating that the control film undergoes severe decomposition during thermal ageing compared to the target film.

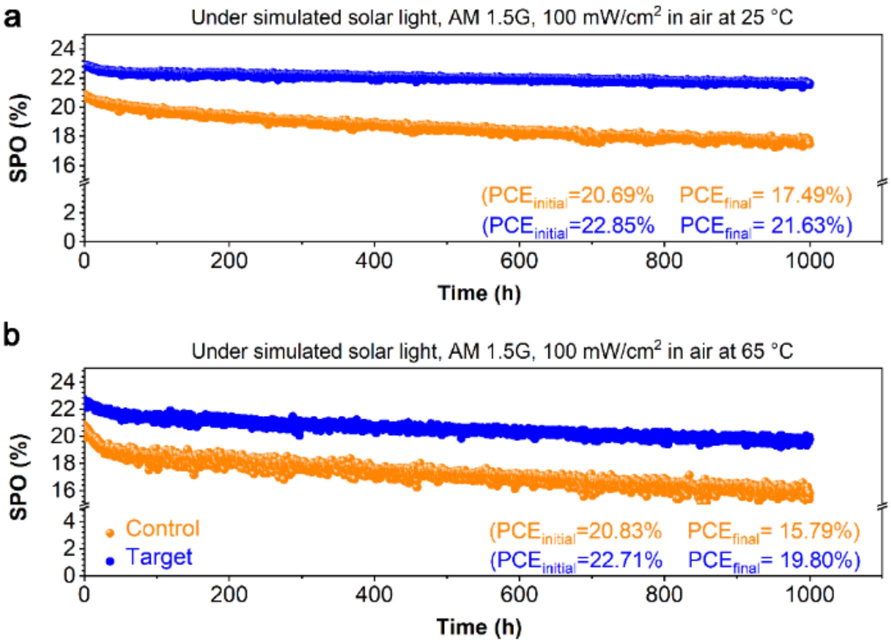

Supplementary Fig. S33 | Stability of the control and target PSMs over 1000 h. (a) ISOS-L-1 stability tests, where the measurement protocol tracks the perovskite module PCE under simulated solar light (AM 1.5G, 100 mW/cm^2) at room temperature. (**b**) ISOS-L-2 stability tests, where the measurement protocol tracks the perovskite module PCE under simulated solar light (AM 1.5G, 100 mW/cm^2) at 65 °C.

Supplementary Tables

Supplementary Table S1 | Previously reported certified perovskite solar modules with active area > 20 cm^2.

Note: PCE refers to the stabilised efficiency measured from quasi-steady-state efficiency or from maximum power point tracking efficiency. ^{ap}, aperture area; ^{da}, designated area; ITO, indium tin oxide; PTAA, poly[bis(4-phenyl)(2,4,6-trimethylphenyl)amine]; P3HT, poly(3-hexylthiophene-2,5-diyl) regioregular; BCP, bathocuproine.

**Device structure**	**Area (cm^2)**	**I_{SC} (mA)**	**V_{OC} (V)**	**FF (%)**	**Stabilised PCE (%)**	**Reverse PCE (%)**	**Time (min)**	**Test center**	**Ref.**
ITO/PTAA/$FA_{0.92}Cs_{0.08}PbI_3$/C_{60}/BCP/Cu	29.5^{ap}	83.6	8.72	75.4	18.6	—	11.5	NREL	6
	44.4^{ap}	81.2	12.91	76.2	18.0	—	15.4		
ITO/PTAA/$MA_{0.6}FA_{0.4}PbI_3$/C_{60}/BCP/Cu	18.1^{ap}	77.0	5.81	78.0	19.25	—	12.8	NREL	7
	50.0^{ap}	76.3	16.1	78.0	19.15	—	14.0		
FTO/c-TiO_2/m-TiO_2/$(FAPbI_3)_{0.95}(MAPbBr_3)_{0.05}$/Spiro-MeOTAD /Au	31.0^{ap}	70.1	11.68	73.8	17.53	19.51	20.0	Newport	8

ITO/SnO_2/$Cs_{0.05}FA_{0.54}MA_{0.41}Pb(I_{0.98}Br_{0.02})_3$/Spiro-MeOTAD-P3HT/Au	22.3[da]	—	—	—	13.88	14.50	7.0	JET	9
—	19.3[da]	64.5	8.04	79.6	21.4	—	—	JET	10
ITO/SnO_2/$(FAPbI_3)_{0.95}(MAPbBr_3)_{0.05}$/$CsPbBr_3$ QD/Spiro-MeOTAD /Au	17.1[ap]	64.6	6.44	72.5	17.85	17.60	1.0	NIM	11
ITO/PTAA/Al_2O_3/$Cs_{0.05}(FA_{0.9}MA_{0.1})_{0.95}Pb(I_{0.9}Br_{0.1})_3$ /C_{60}/SnO_2/Cu/Ag	21.1[ap]	—	—	—	20.1	21.07	5.0	NPVM	12
FTO/c-SnO_2/$CsFAMAPb(I_{1-x}Br_x)_3$/Spiro-MeOTAD/Au	17.1[ap]	65.1	6.99	73.8	19.5	19.6	1.0	NIM	13
ITO/PTAA:BCP/$CsFAPbI_3$/C_{60}/BCP/Cu	26.9[ap]	76.0	9.38	79.5	21.1	21.8	17.8	NREL	14
**FTO/SnO_2@TiO_2/PMMA: PCBM/$Cs_{0.05}MA_{0.05}FA_{0.9}Pb(I_{1-x}Br_x)_3$/PEAI/Spiro-MeOTAD :PDCBT/MoO_3/Cr/Au**	**27.22[ap]**	**83.96**	**9.40**	**79.26**	**22.97**	**23.30**	**5.0**	**NPVM**	**This work**

Supplementary Table S2 | List of NMR control experiments with the corresponding probed effect and observed results.

Entry	Control	Effect tested	Observed result
1	MACl + LiCl	Effect of Cl^- on MA^+ NH peak	Downfield peak shift
2	MACl + HCl	Effect of pH and Cl^- on MA^+ NH peak	Peak broadening, slight downfield peak shift
3	MACl + TFA	Effect of pH on MA^+ NH peak	Peak broadening
4	MACl + LiCl + [Bcmim]Cl	Effect of Cl^- on MA^+ NH peak when [Bcmim]Cl is also present	Peak broadening, downfield peak shift
5	MACl + HCl + [Bcmim]Cl	Effect of pH and Cl^- on MA^+ NH peak when [Bcmim]Cl is also present	Peak broadening, downfield peak shift
6	MACl + $[Bcmim]BF_4$	Effect of $[Bcmim]^+$ on MA^+ NH peak	Peak broadening, slight upfield peak shift
7	MAI + [Bcmim]Cl	Whether MACl and MAI display the same behaviour with [Bcmim]Cl	Peak broadening, downfield peak shift

Supplementary Table S3 | Temperature-corrected pH measurements of ionic liquid solutions (0.0075 M in H_2O, corresponds to the target perovskite precursor solution concentration).

**Ionic liquid**	**pH**
[Dmim]Cl	5.768
[Bmim]Cl	6.095
[Cmmim]Cl	6.107
[Bcmim]Cl	4.826
[Bcmim]BF_4	5.454
[Bcmim]TFSI	5.938

Supplementary Table S4 | Fitting parameters of TRPL spectra and corresponding carrier diffusion length on quartz glass and FTO/*c*-TiO_2/*m*-TiO_2 substrates.

Samples		A_1	τ_1 (ns)	A_2	τ_2 (ns)	L_D (μm)
Quartz glass	Control	0.62	188.6	0.40	977.3	10.6
	Target	0.33	1526.1	0.33	1526.1	14.4
FTO/c-TiO_2/m-TiO_2	Control	0.67	111.6	0.55	284.6	5.7
	Target	0.45	822.1	0.45	822.1	10.5

Note: A_i and τ_i are the decay amplitude and the decay lifetime, respectively. μ is the charge mobility of perovskite film extracted from TRMC measurement. L_d is the carrier diffusion length of perovskite films. The fast decay (τ_1) component is attributed to the surface recombination, while the slow decay (τ_2) component is ascribed to the recombination occurring in the bulk of perovskite films. For the target films, the fitted τ_1 is equal to the τ_2, indicating a single exponential decay process, which is due to the significantly suppressed surface recombination process. The L_D was estimated from the bulk carrier lifetime and bulk mobility based on Equation (2), where k_B is Boltzmann's constant, T is the sample temperature, and τ is the long lifetime of TRPL spectra.

$$L_D = (\frac{k_B T}{e}\mu\tau)^{1/2} \tag{2}$$

The L_D of target films is longer than that of control films on quartz glass, indicating the defect induced charge loss is suppressed.

Supplementary Table S5 | Crystallographic data for $[Bcmim]_4Pb_3Cl_2I_6 \cdot 2DMF$ and $[Bcmim]PbI_3 \cdot DMF$.

**Compound**	**$[Bcmim]_4Pb_3Cl_2I_6 \cdot 2DMF$**	**$[BCmim]PbI_3 \cdot DMF$**
Formula	$C_{20}H_{28}Cl_2I_6N_{10}O_2Pb_3$	$C_{10}H_{14}I_3N_5OPb$
$D_{calc.}$/ g cm^{-3}	3.018	2.825
μ/mm^{-1}	16.690	13.756
Formula Weight	1894.39	808.15
Colour	colourless	colourless
Shape	prism-shaped	prism-shaped
Size/mm^3	0.42×0.07×0.04	0.40×0.05×0.04
T/K	140.00(10)	140.00(10)
Crystal System	monoclinic	orthorhombic
Space Group	$P2_1/c$	$P2_12_12_1$
a/Å	12.2584(4)	8.03648(19)
b/Å	7.8005(3)	12.4511(3)
c/Å	22.0929(7)	18.9893(5)
α/°	90	90
β/°	99.357(3)	90
γ/°	90	90
V/Å^3	2084.45(12)	1900.13(8)
Z	2	4
Z'	0.5	1
Wavelength/Å	0.71073	0.71073
Radiation type	Mo$K\alpha$	Mo$K\alpha$
Θ_{min}/°	2.711	2.698
Θ_{max}/°	28.281	32.772
Measured Refl's.	23621	32257
Indep't Refl's	5182	6573
Refl's I⩾2σ(I)	4438	6106
R_{int}	0.0377	0.0279
Parameters	218	184
Restraints	50	0
Largest Peak/e Å^{-3}	1.466	0.890
Deepest Hole/e Å^{-3}	-2.183	-0.758
GooF	1.064	1.043
wR_2 (all data)	0.0830	0.0374
wR_2	0.0788	0.0360
R_1 (all data)	0.0377	0.0248
R_1	0.0295	0.0202
CCDC number	**2094975**	**2104454**

References

1 Noel, N. K. *et al.* Interfacial charge-transfer doping of metal halide perovskites for high performance photovoltaics. *Energy Environ. Sci.* **12**, 3063-3073 (2019).

2 Xu, W. D. *et al.* Rational molecular passivation for high-performance perovskite light-emitting diodes. *Nat. Photonics* **13**, 418-424 (2019).

3 Seo, J. Y. *et al.* Ionic liquid control crystal growth to enhance planar perovskite solar cells efficiency. *Adv. Energy Mater.* **6**, 1600767 (2016).

4 Yang, W. T., Zhong, D. M., Shi, M. M., Qu, S. X. & Chen, H. Z. Toward highly thermal stable perovskite solar cells by rational design of interfacial layer. *Iscience* **22**, 534-543 (2019).

5 Song, Z. N. *et al.* Impact of processing temperature and composition on the formation of methylammonium lead iodide perovskites. *Chem. Mater.* **27**, 4612-4619 (2015).

6 Deng, Y. *et al.* Defect compensation in formamidinium-caesium perovskites for highly efficient solar mini-modules with improved photostability. *Nat. Energy* **6**, 633-641 (2021).

7 Chen, S. *et al.* Stabilizing perovskite-substrate interfaces for high-performance perovskite modules. *Science* **373**, 902-907 (2021).

8 Yoo, J. W. *et al.* Efficient perovskite solar mini-modules fabricated via bar-coating using 2-methoxyethanol-based formamidinium lead tri-iodide precursor solution. *Joule* **5**, 2420-2436 (2021).

9 Liu, Z. *et al.* A holistic approach to interface stabilization for efficient perovskite solar modules with over 2,000-hour operational stability. *Nat. Energy* **5**, 596-604 (2020).

10 Green, M. A. *et al.* Solar cell efficiency tables (Version 60). *Prog. Photovolt. Res. Appl.* **30**, 687-701 (2022).

11 Zhang, S. *et al.* Improved performance and stability of perovskite solar modules by interface modulating with graphene oxide crosslinked $CsPbBr_3$ quantum dots. *Energy Environ. Sci.* **15**, 244-253 (2022).

12 Gao, Y. *et al.* Can nanosecond laser achieve high-performance perovskite solar modules with aperture area efficiency over 21%? *Adv. Energy Mater.* **12**, 2202287 (2022).

13 You, S. *et al.* Radical polymeric p-doping and grain modulation for stable, efficient perovskite solar modules. *Science* **379**, 288-294 (2023).

14 Fei, C. *et al.* Lead-chelating hole-transport layers for efficient and stable perovskite minimodules. *Science* **380**, 823-829 (2023).
